# Comparison of three serological chemiluminescence immunoassays for SARS-CoV-2, and clinical significance of antibody index with disease severity

**DOI:** 10.1371/journal.pone.0253889

**Published:** 2021-06-29

**Authors:** Nuri Lee, Seri Jeong, Min-Jeong Park, Wonkeun Song

**Affiliations:** Department of Laboratory Medicine, Kangnam Sacred Heart Hospital, Hallym University College of Medicine, Seoul, South Korea; Waseda University: Waseda Daigaku, JAPAN

## Abstract

**Background:**

The clinical significance of the quantitative value of antibodies in severe acute respiratory syndrome coronavirus 2 (SARS-CoV-2) infection remains mostly unidentified. We investigated the dynamics and clinical implications of the SARS-CoV-2 antibody over time using three automated chemiluminescence immunoassays targeting either nucleocapsids or spikes.

**Methods:**

A total of 126 specimens were collected from 23 patients with confirmed and indeterminate COVID-19 identified by molecular tests. SARS-CoV-2 antibody index was measured using SARS-CoV2 IgG reagent from Alinity (Abbott) and Access (Beckman Coulter) and SARS-CoV2 Total (IgG + IgM) from Atellica (Siemens).

**Results:**

Three immunoassays showed strong correlations with each other (range of Pearson’ s correlation coefficient (r) = 0.700–0.854, *P* < 0.001). Eleven (8.7%) specimens showed inconsistencies. SARS-CoV-2 IgG showed a statistically significantly higher value in patients with severe disease than that in non-severe disease patients (*P* < 0.001) and was significantly associated with clinical markers of disease severity.

**Conclusion:**

The quantitative value of the SARS-CoV-2 IgG antibody measured using automated immunoassays is a significant indicator of clinical severity in patients with COVID-19.

## Introduction

Severe acute respiratory syndrome coronavirus 2 (SARS-CoV-2) infection is spreading rapidly worldwide, with high mortality and infection rates. According to the World Health Organization (WHO) dashboard, as of December 24, 2020, 76,858,506 infections and 1,711,498 deaths have been reported worldwide. People infected with SARS-CoV-2 mainly show respiratory symptoms and fever [1] and are associated with pulmonary fibrosis [2] and systemic inflammatory diseases in children [3]. In addition, severe infections in high-risk patients are associated with a mortality rate of 2.7% in men and 1.8% in women [4].

Currently, laboratory tests using molecular diagnostic methods are being conducted for the early diagnosis and isolation of infected persons. However, molecular diagnostic tests demonstrate a high sensitivity in the early stages of the infection, but the detection rate decreases with time. In addition, no information on the patient’s immunity to SARS-CoV2 infection is currently available [5, 6]. To compensate for these limitations, a number of SARS-CoV-2 antibody tests have been developed, but these tests have not yet been sufficiently evaluated and have not yet been applied in actual clinical practice. In addition, serum IgG/M tests have been mainly reported using enzyme immunoassay (ELISA) or rapid diagnostic kits [7]. Both test methods have drawbacks in clinical application. Since ELISA tests are not automated, it is difficult to obtain results within an appropriate time period, and rapid diagnostic kits have the disadvantages of accuracy and difficulty in handling large quantities of tests. Therefore, it is necessary to evaluate the clinical usefulness of the SARS-CoV-2 antibody test using automated equipment.

Several antibody tests currently being developed are designed to detect various regions of the SARS-CoV-2 virus antigen [8]. Since the antigen detection site is different for each antibody test as designed by the manufacturer, there may be differences in the performance of each test. Comparative evaluations for each test are necessary to identify the advantages and disadvantages of each manufacturer’s kit and to select an appropriate kit according to the patient’s underlying disease. In addition, the interpretation of quantitative antibody results (S/CO index) derived through each test has not been evaluated, and the clinical significance of the quantitative value needs to be analyzed. Therefore, in this study, several SARS-CoV-2 antibody tests targeting the nucleocapsid or spike were compared to evaluate the characteristics and differences of each test. In addition, we would like to analyze the trend of changes in the antibody index of patients over time and their clinical significance.

## Materials and methods

### Patients

From June 2020 to November 2020, residual serum samples were collected from patients with confirmed/indeterminate results of COVID-19 molecular tests. Confirmed COVID-19 cases were defined as those that tested positive for SARS-CoV-2 RNA using real-time reverse transcription-polymerase chain reaction (RT-PCR) testing of combined nasopharyngeal and throat swab samples. Indeterminate cases were defined as patients with Ct values between 35 and 40. For patients with confirmed/indeterminate COVID-19, possible follow-up residual samples were also collected, and samples with insufficient quantities were excluded. A total of 126 specimens were collected from the 23 patients. For all specimens, 0.3 mL was aliquoted into microtubes and stored at -70°C before testing. The median number of samples per patient was 6.0 (95% CI = 3.35–6.0), and the median follow-up period was 17 days (95% CI = 15–21). This study was approved by the Institutional Review Board (IRB) of Kangnam Sacred Heart Hospital of Hallym University (IRB No. 2020-08-004-003). Since the anonymity of personal information was maintained, the need for informed consent was waived.

### SARS-CoV-2 antibody testing

The SARS-CoV-2 antibody was measured using three automated serological chemiluminescence immunoassays. The Alinity SARS-CoV-2 IgG reagent (06R86) from Abbott (IL, USA), Access SARS-CoV-2 IgG reagent (C58961) from Beckman Coulter (CA, USA), and Atellica SARS-CoV-2 Total (IgG + IgM) reagents (11206711 COV2T) from Siemens (IL, USA) were utilized in the study. The characteristics of each test are presented in Table 1. Each of the three tests was performed according to the principle of chemiluminescent microparticle immunoassay (CMIA) and chemiluminescence immunoassay (CLIA), and the target site was the nucleocapsid for Alinity and spike for Access and Atellica. The cut-off indices for each positive immunoassay were 1.4, 1.0, and 1.0, respectively.

**Table 1:** Information on three chemiluminescence immunoassay used for testing antibody of COVID-19.

	Alinity	Access	Atellica
	SARS-CoV2 IgG	SARS-CoV2 IgG	SARS-CoV2 Total
Company	Abbott Laboratories (IL, USA)	Siemens Healthcare Diagnostics (IL, USA)	Beckman Coulter Inc. (Brea CA, USA)
Targeting antibody	IgG	IgG	Total antibody (including IgG and IgM)
Protein targeting	Nucleocapsid	Spike	Spike
Methodology	chemiluminescent microparticle immumoassay	chemiluminescence immunoassay	chemiluminescence immunoassay
Specimen type (s)	Serum, plasma	Serum, plasma	Serum, plasma
Required specimen amount	75 μL (Sample volume for each additional test from same sample cup: 25 μL)	20 μL	50 μL
Interpretation of results	Cut-off index (Sample/calibrator, S/C), < 1.4: Negative; ≥ 1.4: Positive	Cut-off index (S/CO), ≤0.80: Non-reactive; <0.8 to <1.0: Equivocal; ≥ 1.0: Reactive	Cut-off index (Using the calculation procedure in the system), <1.0: Non-reactive; ≥ 1.0: Reactive

### Statistics

Pearson’s correlation coefficient was used to analyze the correlation between multiple SARS-CoV-2 antibody tests and clinical features. The degree of correlation was weak for values belonging to the range 0.10 ≤ r <0.30, moderate for values of 0.30 ≤ r <0.50, and strong correlation for r ≥ 0.50, as previously guided [9]. The Mann-Whitney U test was conducted to evaluate the statistical significance of the difference in clinical aspects according to the SARS-CoV-2 IgG S/CO value. Statistical analyses were performed using SPSS version 24 (IBM Corporation, New York, NY) and MedCalc version 18 (MedCalc Software, Mariakerke, Belgium).

## Results

### Characteristics of patients

Of the 23 patients enrolled in the study, 5 with a severe clinical course and 18 with no severe clinical course were divided into two groups and compared. Patients with severity were defined as those who showed pneumonia and had been intubated at least once during hospitalization and used a ventilator. Table 2 summarizes the laboratory test results and demographic data at the time of admission. In the severe patient group, the median age was 72.0 years (Interquartile range (IQR): 70.8–77.3), which was statistically significantly higher than that of the non-severe group (63.5 years, IQR: 59.0–72.0). Underlying diseases, including hypertension and diabetes, were present in all patients in the severe group. In the non-severe group, nine participants were healthy, and the remaining nine had underlying diseases. Two (40.0%) patients in the severe patient group died. There was no statistically significant difference between the complete blood count (CBC) and chemistry panel performed at the time of admission. The median CRP level was 133.9 mg/dL (IQR: 80.8–139.7) in the severe group and significantly higher than 18.3 mg/dL (IQR: 2.9–55.7) in the non-severe group. The median values of the SARS-CoV-2 S/CO index at admission, measured using three antibody test kits, were 0.37, 0.46, and 0.57, respectively. Hepatitis serology (HBsAg, anti-HBs, and anti-HCV), RPR test for syphilis, and HIV antibody information on the day of hospitalization were investigated. None of the patients were positive for syphilis, HIV, or HCV; 12 (52.2%) patients were positive for anti-HBs, while one patient tested positive for HBsAg.

**Table 2:** Demographic laboratory characteristics of patients on admission.

	All	Non-severe	Severe^⊃^
	(N = 23)	(N = 18)	(N = 5)
Age, year	67.0 (60.0–72.0)	63.5 (59.0–72.0)	72.0 (70.8–77.3)*
Gender (Male:Female)	16:7	12:6	4:1
Underlying disease			
None/HTN/DM/HF/Respiratory/Other	9/8/5/1/2/4	9/5/3/1/1/3	0/3/2/0/1/1*
Presenting symptoms			
Fever/Cough/Pneumonia/None/Other	9/6/9/4/5	4/5/4/4/4	5/1/5/0/1
Seroconversion days	10.0 (7.0–12.0)	10.0 (5.5–11.8)	10.5 (7.5–14.5)
Duration of ICU stay (Days)	16.0 (10.8–20.5)	16.0 (10.8–19.3)	28.0 (12.5–42.5)
Death (Yes:No)	2:21	0:18	2:3**
rRT-PCR, Ct	26.0 (18.5–31.7)	25.7 (17.9–31.8)	27.2 (26.3–30.5)
No. of antibody tested sample	126	98	28
**Blood tests on admission**			
Hemoglobin (g/dL)	13.3 (13.0–14.9)	13.7 (13.0–15.5)	13.0 (11.9–13.3)
Total white blood cell count (× 10^9^/ L)	5.13 (3.81–7.05)	5.13 (3.71–5.84)	8.2 (4.55–10.25)
Absolute neutrophil count (× 10^9^/ L)	3.57 (2.29–4.62)	2.99 (2.24–4.07)	6.58 (3.51–8.74)
Lymphocyte count (× 10^9^/ L)	0.86 (0.69–1.26)	0.98 (0.75–1.38)	0.67 (0.57–0.88)
Platelet (× 10^9^/ L)	163.0 (148.3–224.8)	207.5 (148.0–253.0)	156.0 (142.3–160.0)
Creatinine (μmol/L)	0.74 (0.61–0.84)	0.75 (0.60–0.88)	0.73 (0.57–0.79)
Aspartate aminotransferase, U/L	35.0 (28.5–45.8)	33.0 (24.0–43.0)	55.0 (51.0–64.0)**
Alanine aminotransferase, U/L	29.0 (18.3–35.0)	28.0 (17.0–34.0)	35.0 (20.5–41.5)
Procalcitonin (ng/mL)	0.03 (0.03–0.07)	0.03 (0.03–0.05)	0.05 (0.03–0.17)
CRP (mg/dL)	31.0 (3.2–119.3)	18.3 (2.9–55.7)	133.9(80.1–139.7)*
**SARS-CoV-2 Antibody Index**			
Alinity CoV-2 IgG (S/CO)	0.37 (0.03–5.88)	0.53 (0.028–5.963)	0.20 (0.650–4.085)
Access CoV-2 IgG (S/CO)	0.46 (0.11–13.17)	0.47 (0.103–18.255)	0.15 (0.093–24.890)
Atellica CoV-2 Total (S/CO)	0.57 (0.348–10.0)	0.49 (0.315–10.0)	0.66 (0.540–3.205)

Continuous data are quoted as median values with interquartile range.

*P < 0.05

**P < 0.005 Data are compared using a Mann-Whitney or Chi-square test between “severe” and “non-severe”.

⊃Severe patients are included follow criteria: pneumonia, intubation.

Abbreviation: HTN, hypertension; DM, diabetes mellitus; HF, heart failure.

### Comparison of serological chemiluminescence immunoassays

**Fig 1:**
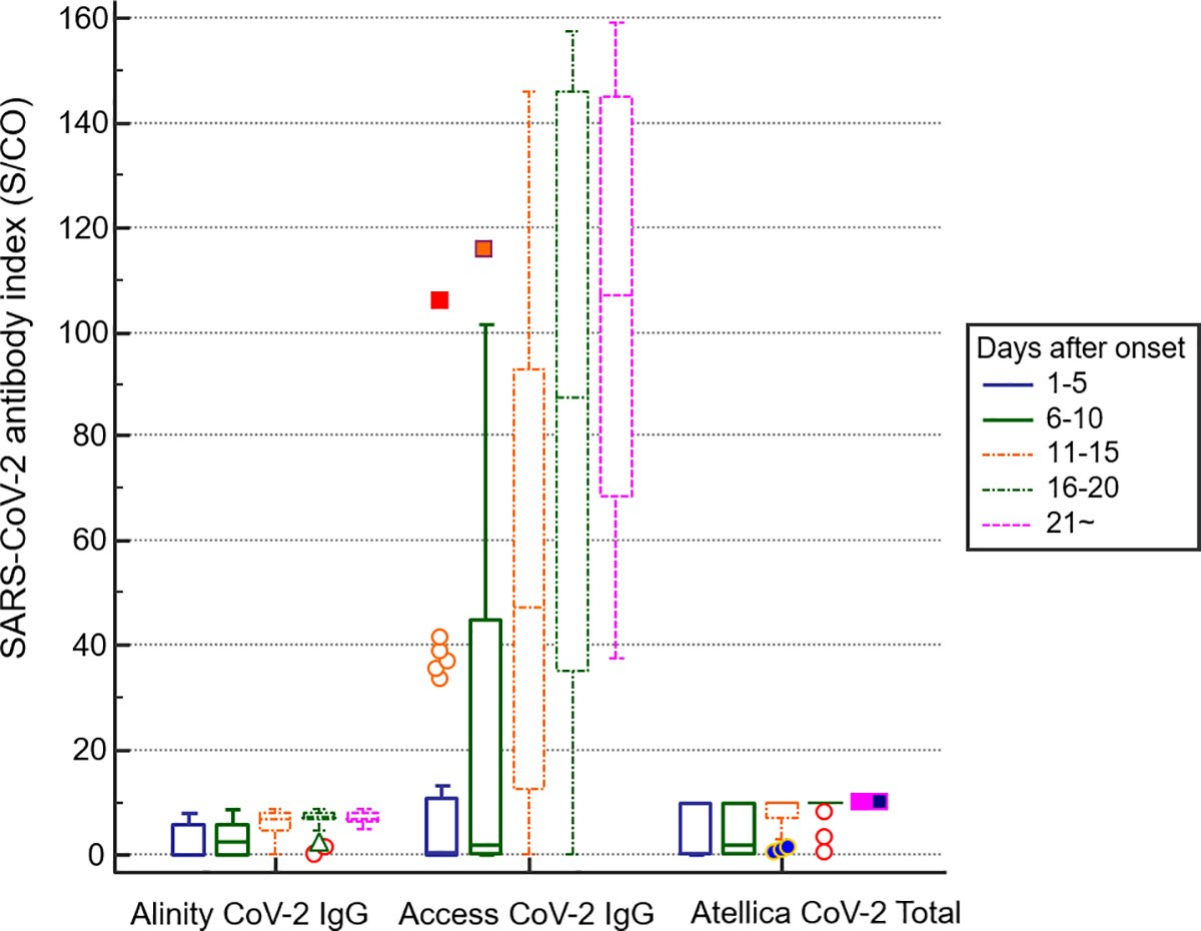
Distribution of the SARS-CoV-2 antibody S/CO index measured by three chemiluminescence immunoassays according to days after onset.

**Fig 2:**
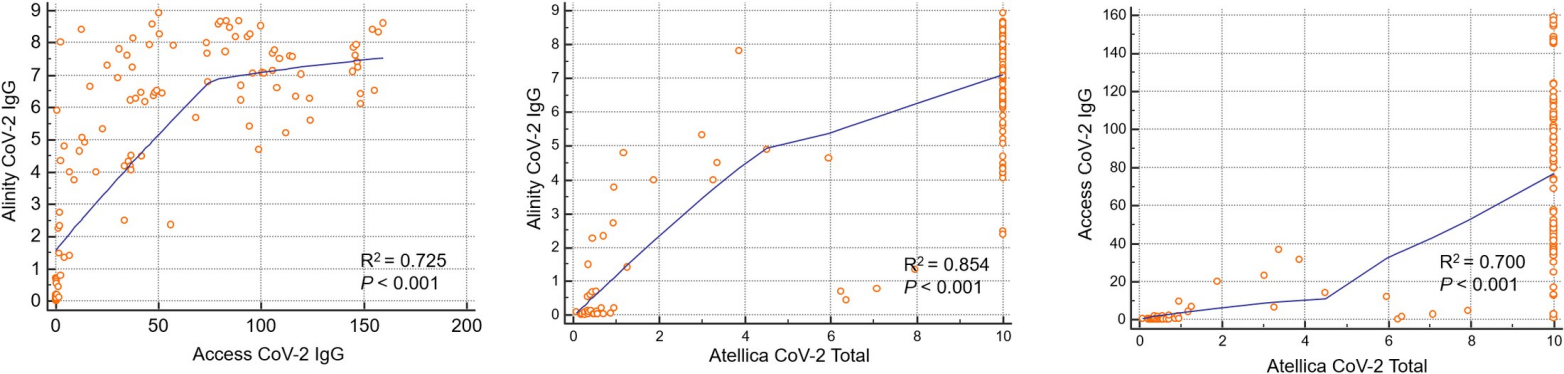
Pearson’s correlation coefficient r and p-value between S/CO index of the three automated immunoassays. Alinity CoV-2 IgG, Access CoV-2 IgG, and Atellica CoV-2 Total antibody presented strong correlations.

Alinity was tested in all specimens out of a total of 126 specimens. Access was tested in 123 specimens, and Atellica was tested in 120 specimens. When measured by Alinity, a total of 93 samples (73.8%) were positive, and 33 samples (26.2%) were negative. In the case of Access, 93 samples (73.8%) were positive, 29 samples (23.0%) were negative, and one sample (0.8%) showed equivocal results. In the results of Atellica, 89 samples (70.6%) were positive, and 31 samples (24.6%) were negative. Median values for each instruments were 6.21 (IQR: 1.2–7.6) for Alinity, 37.02 (IQR: 1.4–94.2) for Access, and 10.0 (IQR: 0.96–10.0) for Atellica (Fig 1). Alinity, Access, and Atellica showed strong correlations with each other (Fig 2). For the value of Pearson’ s correlation coefficient r, Alinity and Access were 0.725 (*P* < 0.001), Access and Atellica 0.6997 (*P* < 0.001), and Alinity and Atellica were 0.854 (*P* < 0.001).

**Fig 3:**
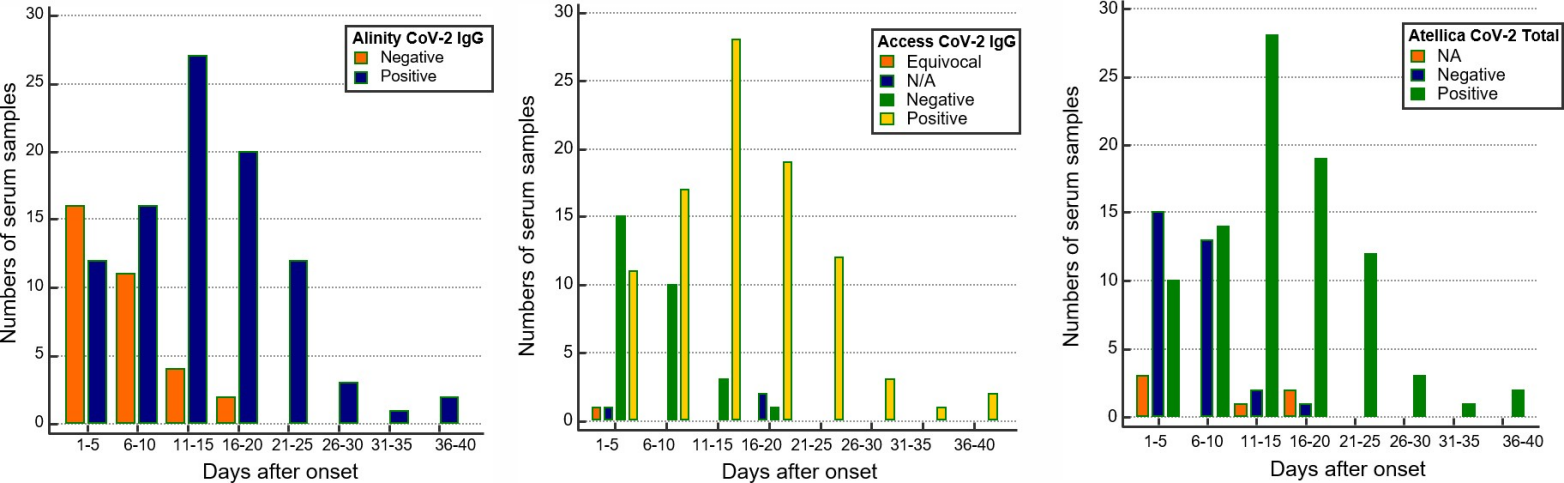
Samples with SARS-CoV-2 antibody levels at different time points after the onset of symptoms from patients who showed confirmed/indeterminate results of COVID-19 molecular tests. (A) Alinity CoV-2 IgG, (B) Access-CoV-2 IgG, and (C) Atellica CoV-2 Total antibody presented positive in all specimens after 21 days from symptom onset.

Fig 3 shows the number of positive and negative samples for SARS-CoV-2 antibody measured with the three immunoassays over time from the onset of symptoms. Specimens collected after 21 days were positive for all the equipment. Two specimens were negative between and 16 and 20 days, one of which was negative only in Alinity, and the other was negative in all three devices. In this patient, seroconversion was observed on the 12^th^ day, but the tests performed on the 18^th^ day showed negative results (S1 Fig, Patient 13).

### Discrepancy of the positivity of SARS-CoV-2 antibody

**Fig 4:**
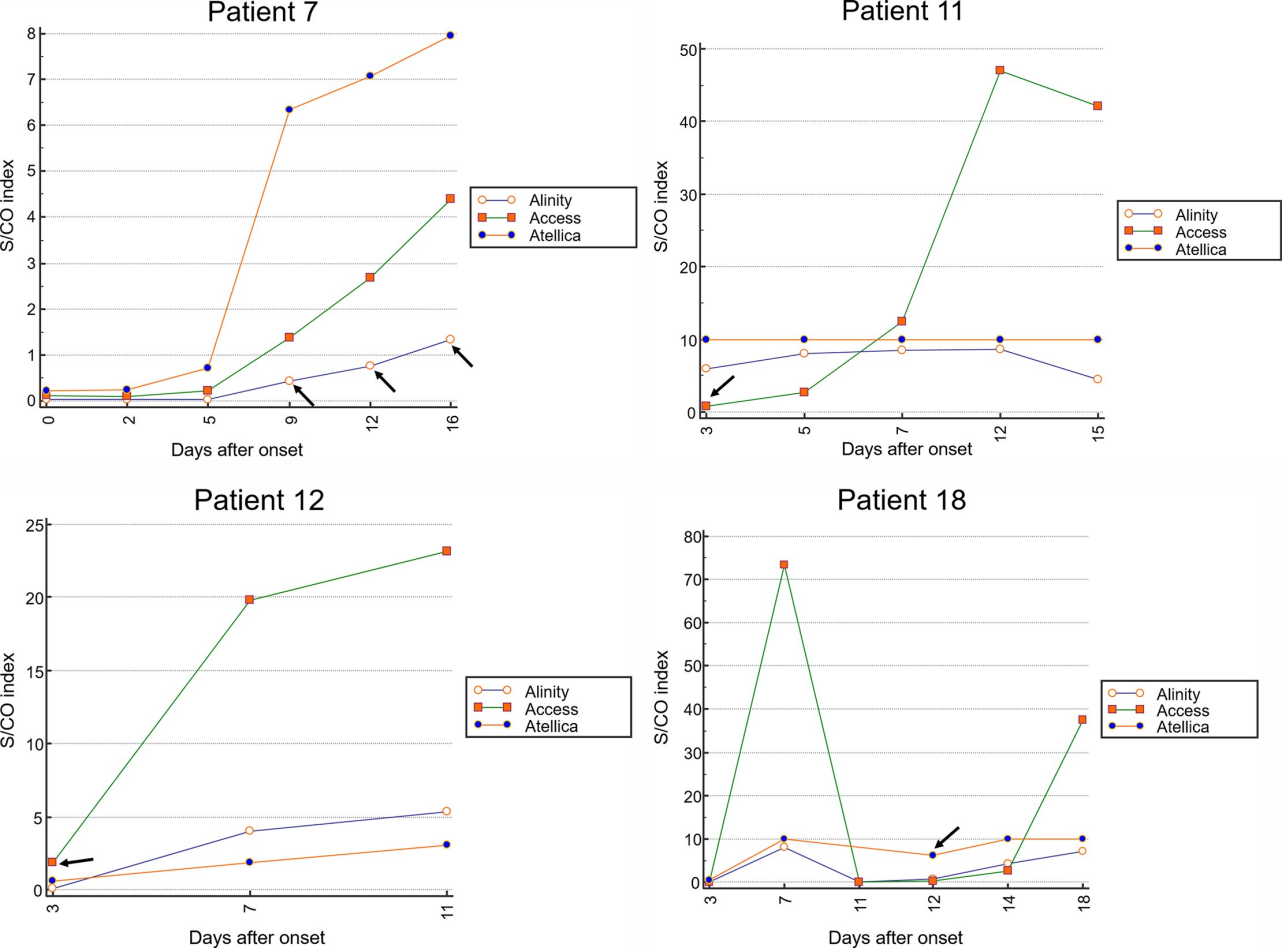
Dynamic profiling of serum SARS-CoV-2 antibody measured by three chemiluminescence immunoassays. The black arrow indicates the specimens showing inconsistency among the three equipment. (A) Alinity CoV-2 IgG on days 9, 12, and 16 was negative, and Access/Alinity showed positive on the same specimens. Only the specimens measured by Access on the 3^rd^ day after symptom onset was negative (B) and positive (C). (D) Atellica CoV-2 Total antibody was positive on the 12^th^ day, whereas the other two reagents were negative on the same specimen.

Of the 126 specimens, 121 were tested with all equipment, and 11 (8.7%) showed discrepancies. Table 3 provides additional information on specimens showing inconsistent results. Three specimens were negative only in Alinity, and all of them were collected from one patient (Patient 7) on the 9^th^, 12^th^, and 16^th^ days after symptom onset. In this patient, specimens at 1, 2, and 5 days of symptoms presented were all negative by the three instruments (Fig 4A). In patient 11, only the specimens measured by Access were equivocal, and the other two instruments showed positive results (Fig 4B). On the contrary, in patient 12, the results that were positive in the other two devices were negative only in Access (Fig 4C). In both cases, the samples were measured three days after symptom onset, which corresponds to the early stage of the symptoms. Patients 8, 15, 21, and 23 showed negative values only in Atellica and positive values in the other two instruments (S1 Fig). For these samples, the median value of the S/CO index measured by Atellica was 0.65, which was close to the cut-off value. The samples measured with the other two instruments also showed a median index of 2.26, which did not deviate much from the cut-off value. In the case of patient 18, only Atellica, which measures the total antibody, was positive (Fig 4D) on day 12^th^.

**Table 3:** Patients showed discrepancy in SARS-CoV-2 antibody positivity in three automated immunoassay system.

Patient	Date of symptoms onset	Date of antibody measure	Days after symptoms onset	Ct value	PCR	Alinity index	Alinity result	Access index	Access result	Atellica index	Atellica result
Patient 7	2020-07-18	2020-07-27	9	28.96	Positive	0.43	Negative	1.38	Positive	6.35	Positive
Patient 7	2020-07-18	2020-07-30	12	28.12	Positive	0.77	Negative	2.68	Positive	7.08	Positive
Patient 7	2020-07-18	2020-08-03	16	N/A	Negative	1.33	Negative	4.38	Positive	7.95	Positive
Patient 8	2020-08-25	2020-09-02	8	27.18	Positive	2.72	Positive	1.95	Positive	0.94	Negative
Patient 11	2020-09-23	2020-09-26	3	20.46	Positive	5.88	Positive	0.81	Equivocal	10	Positive
Patient 12	2020-08-20	2020-08-23	3	25.29	Positive	0.11	Negative	1.83	Positive	0.57	Negative
Patient 15	2020-09-18	2020-09-28	10	20.62	Positive	2.33	Positive	2.26	Positive	0.72	Negative
Patient 15	2020-09-18	2020-09-30	12	N/A	Negative	3.75	Positive	9.37	Positive	0.96	Negative
Patient 18	2020-08-06	2020-08-18	12	25.04	Positive	0.7	Negative	0.16	Negative	6.23	Positive
Patient 21	2020-08-16	2020-08-19	3	N/A	N/A	1.48	Positive	1.72	Positive	0.37	Negative
Patient 23	2020-08-26	2020-09-03	8	N/A	N/A	2.26	Positive	1.45	Positive	0.46	Negative

### Clinical significance of SARS-CoV-2 antibody S/CO index

**Fig 5:**
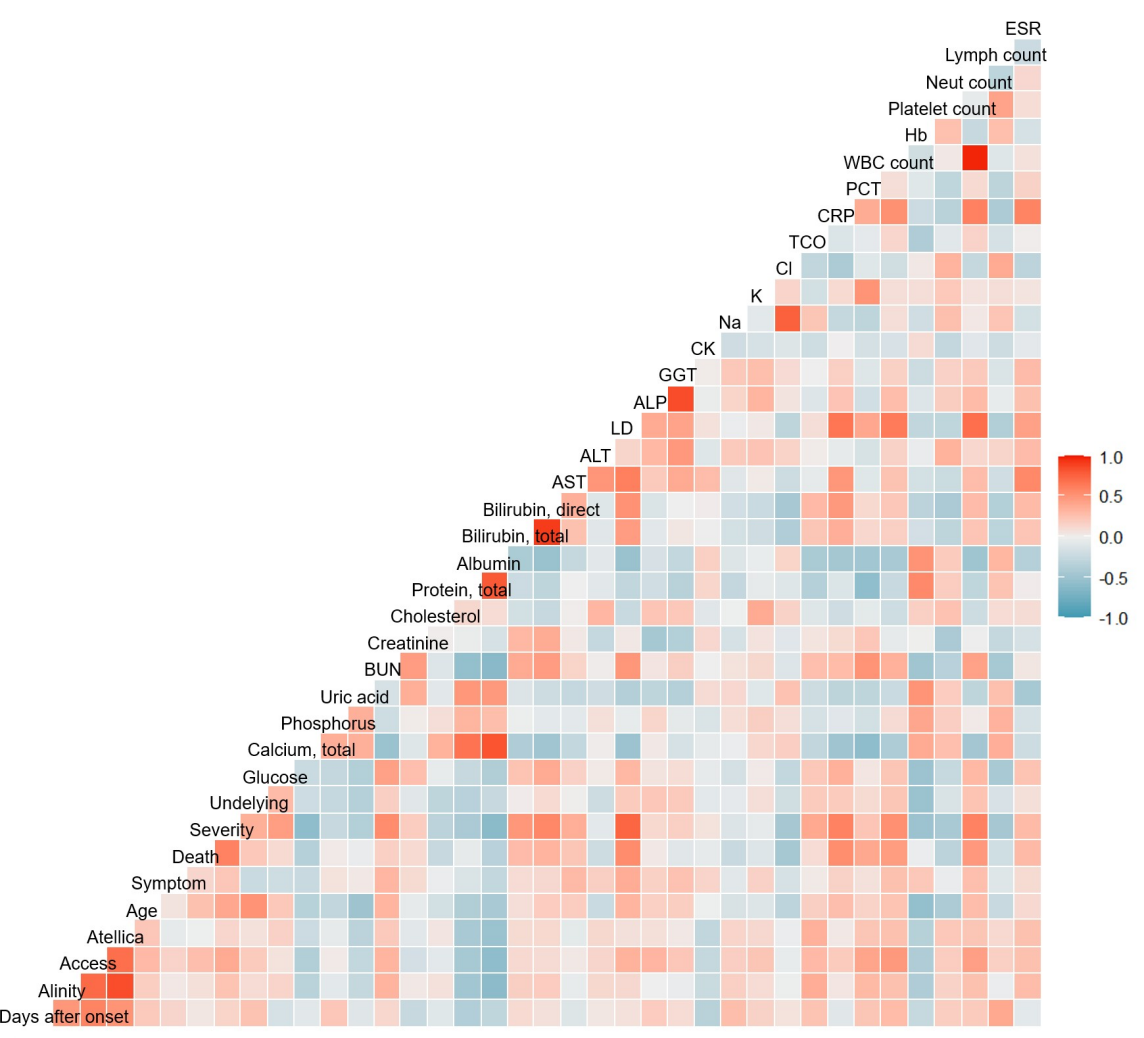
Pairwise association between clinical biomarkers indicating disease severity and SARS-CoV-2 antibody index.

Fig 5 shows the correlation between the clinical biomarkers for disease severity, including the SARS-CoV-2 index value measured by the three automated devices. Moderate positive correlations were observed for WBC (Pearson’s correlation coefficient (r) = 0.483, *P* < 0.001), neutrophil count (r = 0.444, *P* < 0.001), LD (r = 0.341, *P* < 0.001), and BUN (r = 0.328, *P* < 0.001). Statistically significant moderate negative correlations were observed for calcium (r = -0.371, *P* < 0.001), total protein (r = -0.439, *P* <0.001), albumin (r = -0.578, *P* < 0.001), and hemoglobin (r = -0.386, *P* < 0.001).

**Fig 6:**
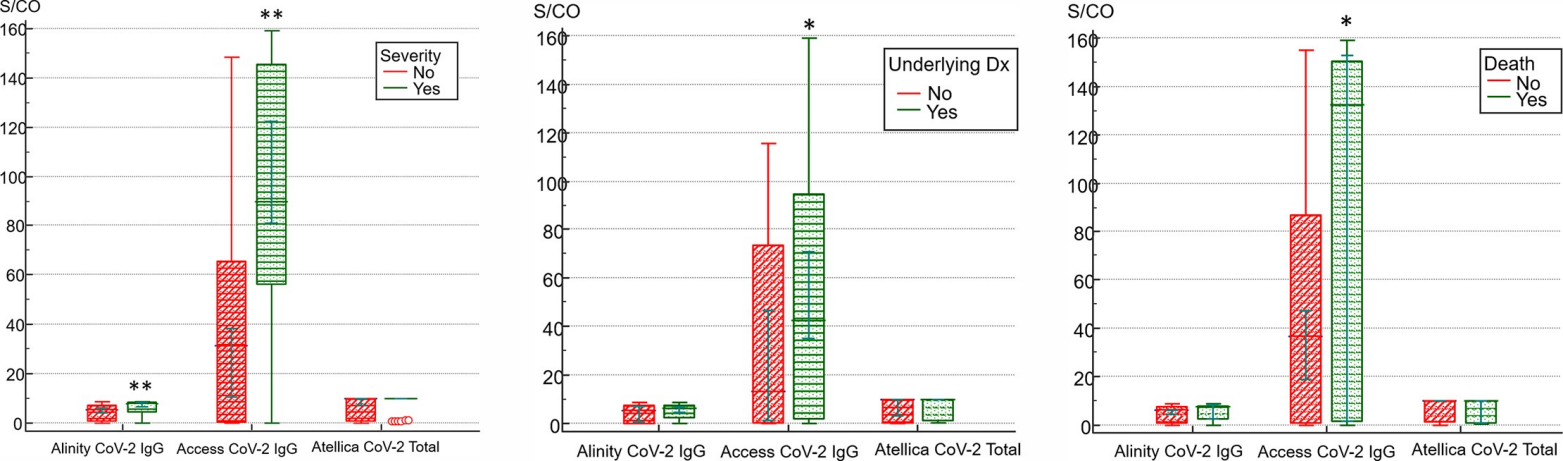
Mann-Whitney’s U comparison between (A) patients with severity and non-severe group (B) patients with and without underlying disease (C) patients classified by mortality. Access CoV-2 IgG antibody had a statistically significantly higher S/CO index in severe patients, patients with underlying disease, and patients who died. Alinity CoV-2 IgG antibody had a significantly more increased S/CO index in severe patients than in those of the non-severe group. *P < 0.05, **P < 0.005.

We analyzed the statistical differences in the antibody index values according to disease severity, underlying disease, and mortality for each instrument (Fig 6). The SARS-CoV-2 IgG antibody measured by Alinity and Access showed significantly higher values in patients with severity (7.79 vs. 5.32, *P* = 0.0002 for Alinity, 89.69 vs. 31.17, *P* = 0.0005 for Access) than that in non-severe patients. In addition, Access to SAR-CoV IgG showed a statistically significantly higher value in patients with underlying disease (*P* = 0.039) and statistically significantly higher values in the deceased patients (*P* = 0.023).

### Dynamics of the SARS-CoV-2 antibody

In the enrolled patients in this study, antibody index was maintained or gradually increased in 14 patients (73.7%) during the study period from 4 to 45 days per patient. Of a total of 23 patients, four with fewer than three follow-up specimens were excluded, and the dynamics of SARS-CoV-2 antibody measured with three instruments for 19 patients are described in the S1 Fig. Patients 2, 8, 10, 17, and 23 showed severe symptoms and demonstrated increased antibody index values that lasted from 16, 8, 11, 7, and 8 days after the onset of symptoms to discharge from the hospital, respectively.

Meanwhile, three patients (Patient 4, 10, and 14), from the onset of symptoms to the time of discharge, showed sustained positive antibody index in all devices. Among them, Patient 4 was positive for HBsAg. In contrast, in five patients, the antibody index decreased during the measurement period. In patients 11, 13, and 20, the antibody levels decreased on the 12^th^, 15^th^, and 20^th^ days from symptom onset, respectively. Patients 17 and 18 showed a pattern of decrease on the 21^st^ and 7^th^ days, respectively, and then increased again on the 42^nd^ and 14^th^ days. Patients showed.

## Discussion

In this study, the SARS-CoV-2 antibody, which is currently emerging worldwide, was measured using three automated chemiluminescence immunoassays, and the clinical significance of the antibody index was analyzed. The three types of immunoassay instruments had a strong correlation with each other and showed a concordance rate of 91.3%. In addition, the quantitative index was significant as a biomarker, reflecting the severity of the patient’s clinical manifestations.

After the pandemic of SARS-CoV-2 infection, the dynamics and performance of the SARS-CoV-2 antibody have been actively studied [10–13]. However, we have limited information, and the results often show inconsistencies. In addition, there are few studies on the correlation between the clinical features of patients and the results of various laboratory tests, including the SARS-CoV-2 antibody. According to a study by S Phipps et al., the antibody index values of IgG and IgM for SARS-CoV-2 cannot reflect or predict disease severity [14]. Gozalbo et al. reported that the SARS-CoV-2 spike protein receptor-binding domain IgG antibody titer and neutralizing antibody levels measured by ELISA were less associated with pro-inflammatory biomarkers [15]. On the other hand, in the study of antibody dynamics conducted by Chen et al., SARS-CoV-2 IgG measured by two chemiluminescence devices showed high quantitative values in patients with pneumonia [16]. Liu and Zhao et al. have also reported that critical patients show higher titers of antibodies [17, 18]. It is considered that differences in quantification methods, specifically disparity in precision, range, and linearity of quantification, could be one of the causes of these discrepancies. In general, it has been reported that CLIA has higher specificity and accuracy than quantitative methods such as ELISA and agglutinin for the detection of many viruses [19–21]. The quantitative values calculated by automated chemiluminescence instruments could be more consistent and wider than those measured by ELISA in earlier studies and would achieve a better correlation with clinical features. There are few studies on the quantification of SARS-CoV-2 antibody using the CLIA or CMIA method so far; as such, more studies on the accuracy and clinical significance of quantitative values should be conducted. With the equipment used in this study, a pilot study of precision tests was performed with SARS-CoV-2-positive control reagent for three instruments and revealed a coefficient of variation percentage for Alinity of 1.5%, Access 2.80%, and Atellica 3.73%. In the follow-up study, a precise evaluation of the equipment and comparison of performance through more specimens should be performed.

The three automated chemiluminescence equipment showed a correlation of 0.700–0.854 with each other, and when analyzing the results showing inconsistency, the discrepancy rate was 8.7%. Among the possible causes of inconsistency, the specimens classified as positive or negative with a quantitative value near the cut-off at the seroconversion period accounted for the most (Patients 8, 15, 21, and 23). Additionally, there was one patient who could not detect the antibody only in the Alinity test, the only device that had a different target protein (Patient 7). Finally, one patient was positive only in the Atellica device. Since this device measures total antibodies, including IgG and IgM, it was assumed that in this patient, IgG was negative and IgM was positive (Patient 18). Regarding the severity of illness in patients in this study, Alinity and Access (measuring for SARS-CoV-2 IgG antibody index), showed a statistically significant correlation, but Atellica (measuring for total antibody index) did not show the same. Measurement of total antibody presented better sensitivity and specificity compared to other immunoassays in previous studies [22]. Therefore, the Atellica SARS-CoV2 Total assay is considered to be more useful as a supplemental assay for diagnosis of SARS-CoV-2 than factors associated with clinical severity.

The strength of this study is that serial follow-up for a relatively long period of time was possible in each patient. The dynamics of the SARS-CoV-2 antibody quantification value over time have been studied with a lot of interest in relation to individual immunity. In this study, 73.7% of patients during the follow-up period, antibody values were either maintained or increased. In 26.3% of patients, antibody index began to decrease on average after 15.4 days (95% CI = 7.61–23.2), and some of these patients showed a pattern of increasing again. In the case of Patient 4, all equipment showed positive SARS-CoV-2 antibodies from the 3^rd^ day to the 20^th^ day of symptoms. The patient was characteristically HBsAg-positive. Previously, the SARS-COV-2 antibody test showed false positives in acute infection [23, 24]. In particular, acute HBV infection showed cross-reactivity with the SARS-CoV-2 antibody assay in 75% of patients. This study also provides an example that HBsAg can cause false-positive results in various automated immunoassays. The other two patients (Patients 10 and 14) also showed high index-positive values throughout the hospitalization period, but the cause could not be determined. Follow-up studies will be needed to characterize patients with sustained or rapidly decreased in antibody index. In addition, many papers have been published on the clinical implications of target proteins in the detection of SARS-CoV-2 antibodies. Sun et al. reported that consistently high nucleotide proteins are associated with disease progression or severe illness, and spike protein has a protective effect on prognosis [25]. However, in this study, a rough estimate of the immunoassay results for each target protein can be measured due to the use of different equipment.

The limitation of this study was the small number of enrolled patients. Nevertheless, it was possible to collect multiple serial samples for each patient, with a follow-up period of a minimum of three days to a maximum of 45 days. In particular, a relatively large number of patients were recruited at the initial stage of symptoms, so the seroconversion period was included in 87.0% of patients. The second limitation of this study was that the quantitative scale was different for each device. In particular, it is difficult to quantitatively compare each target protein. Therefore, it must be considered in the comparative analysis of each device, and as a follow-up study, precise target protein studies using a certain quantitative measurement tool would be required.

As the cumulative number of patients with SARS-CoV-2 infection increases and long-term management is required, the importance of diagnosis, as well as the appropriate care and recovery of patients with high severity, are emphasized. As can be seen from the results of this study, the SARS-CoV-2 antibody has a significant correlation with clinical severity and is an important index reflecting the clinical characteristics of each patient. In particular, the S/CO index value obtained through automated equipment is expected to be actively used in the future as a quantitative value that can be obtained quickly and easily in clinical practice.

## Supporting information

S1 Fig Dynamic profiling of SARS-COV-2 antibody during hospitalization for 19 of the patients enrolled in the study. SARS-COV-2 antibody was measured by three types of automated chemiluminescence immunoassays (Alinity CoV-2 IgG, Access CoV-2 IgG, and Atellica CoV-2 Total).
(DOCX)
