# Supplementary figures and images for: Comparison of three serological chemiluminescence immunoassays for SARS-CoV-2, and clinical significance of antibody index with disease severity
Source: PLoS One. 2021 Jun 29;16(6):e0253889. doi: 10.1371/journal.pone.0253889 (PMC8241106; doi:10.1371/journal.pone.0253889)

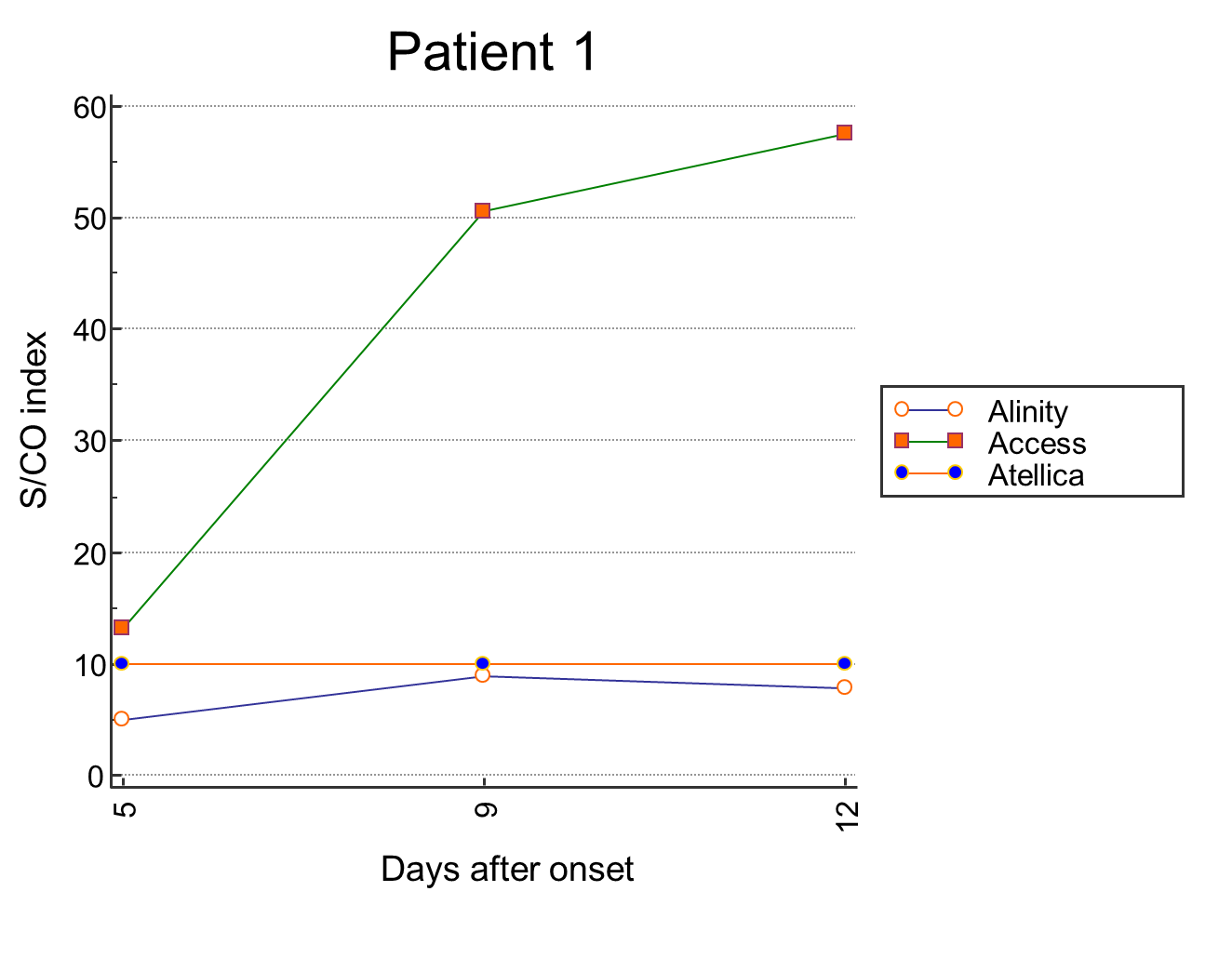
S1 Fig.


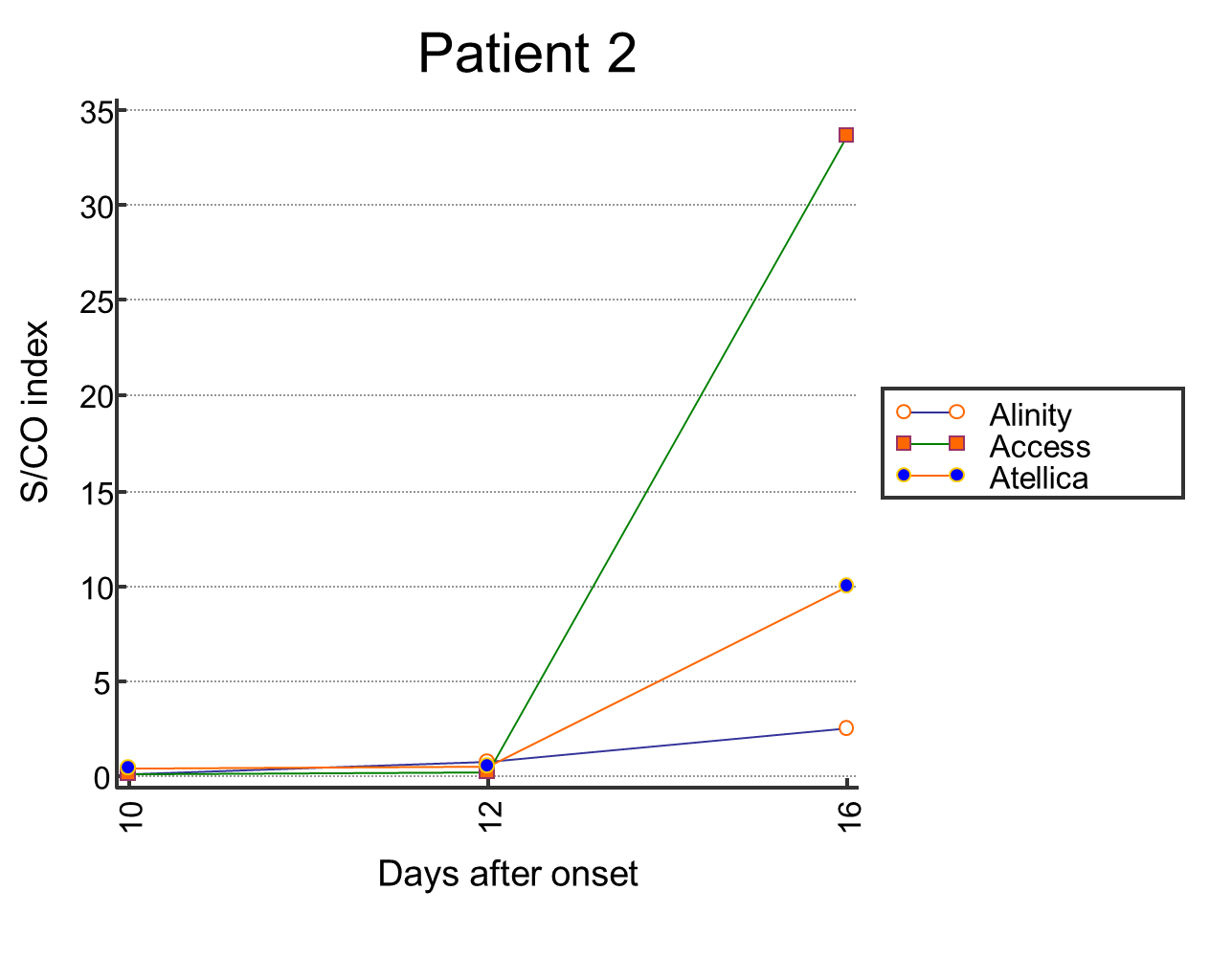


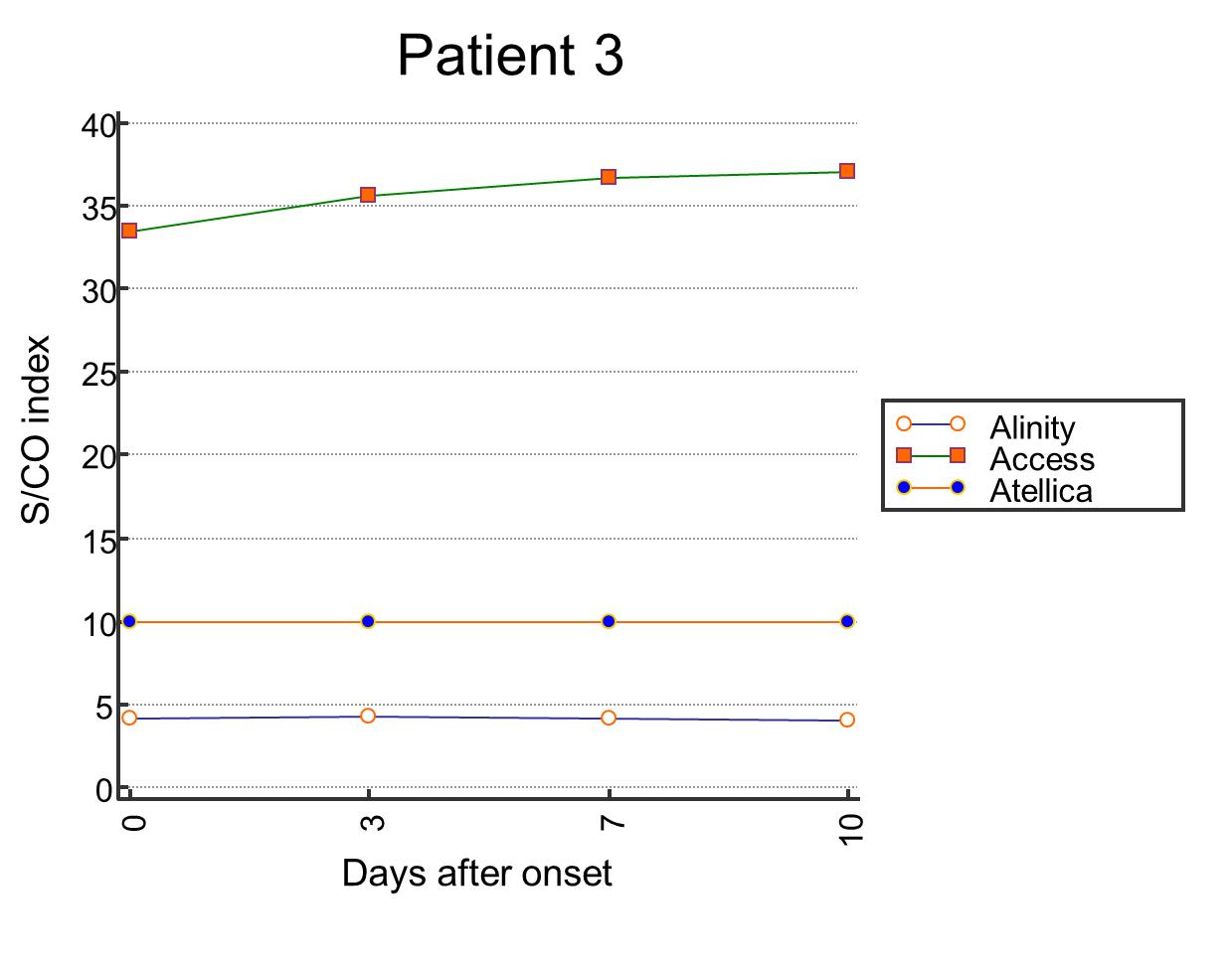

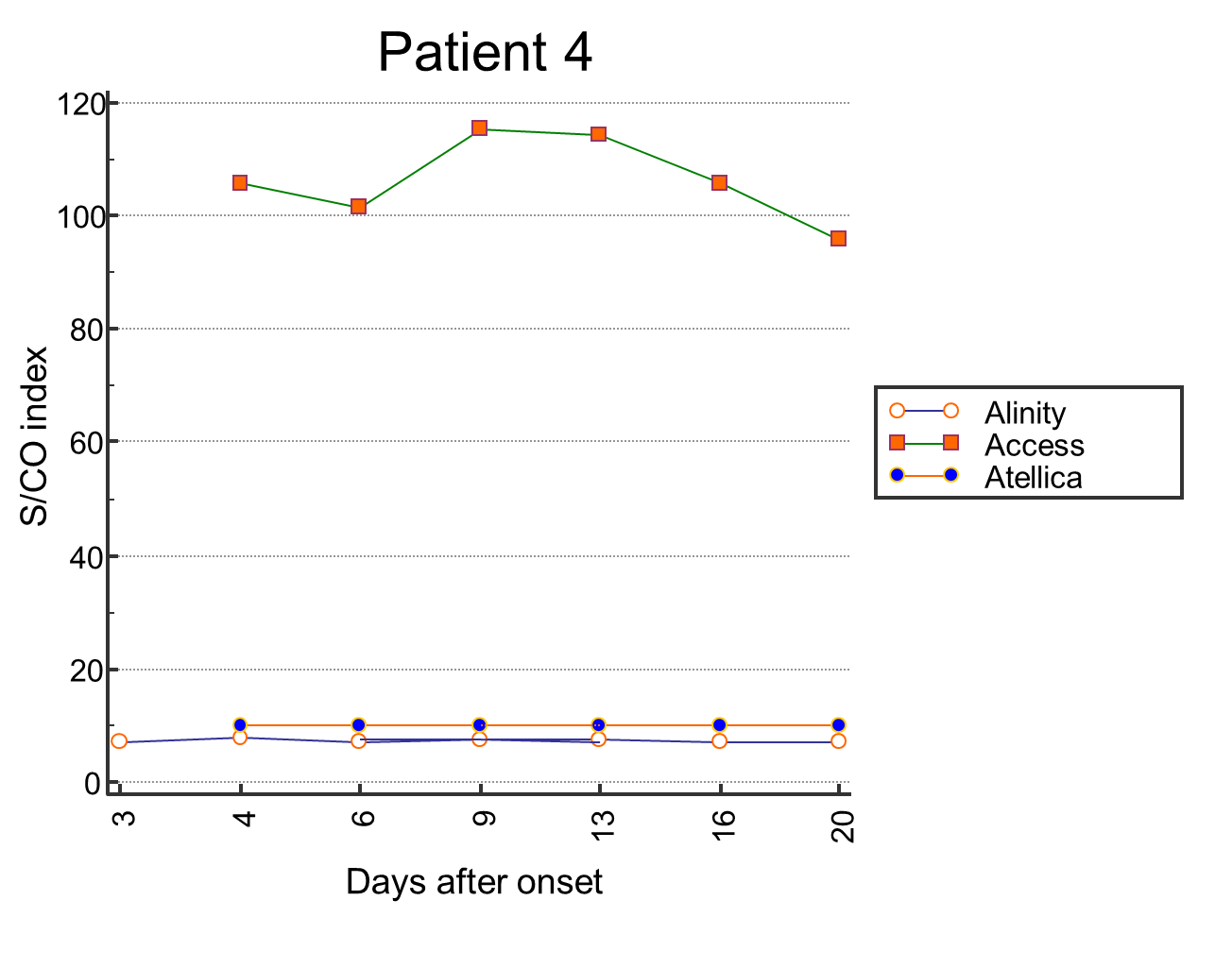

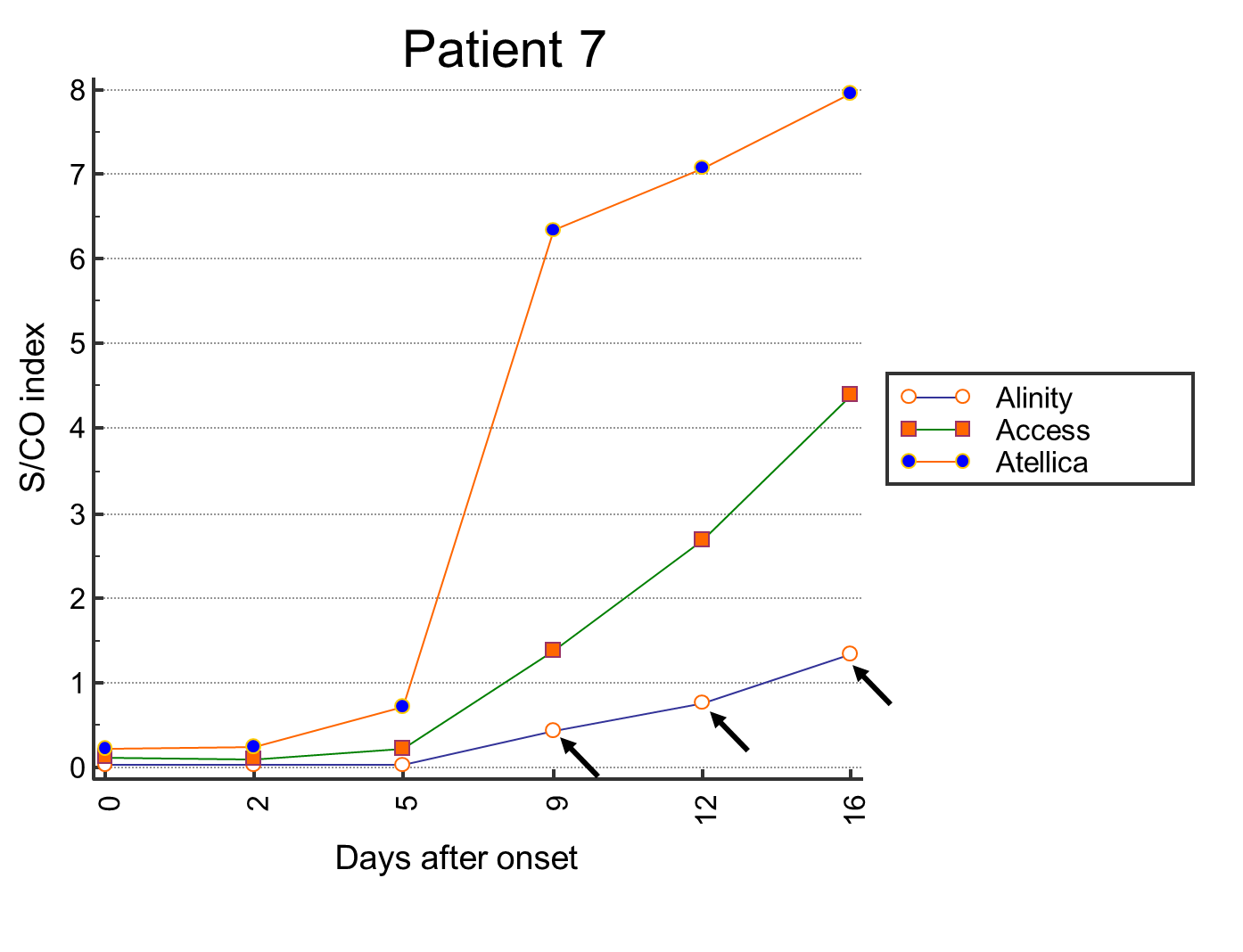

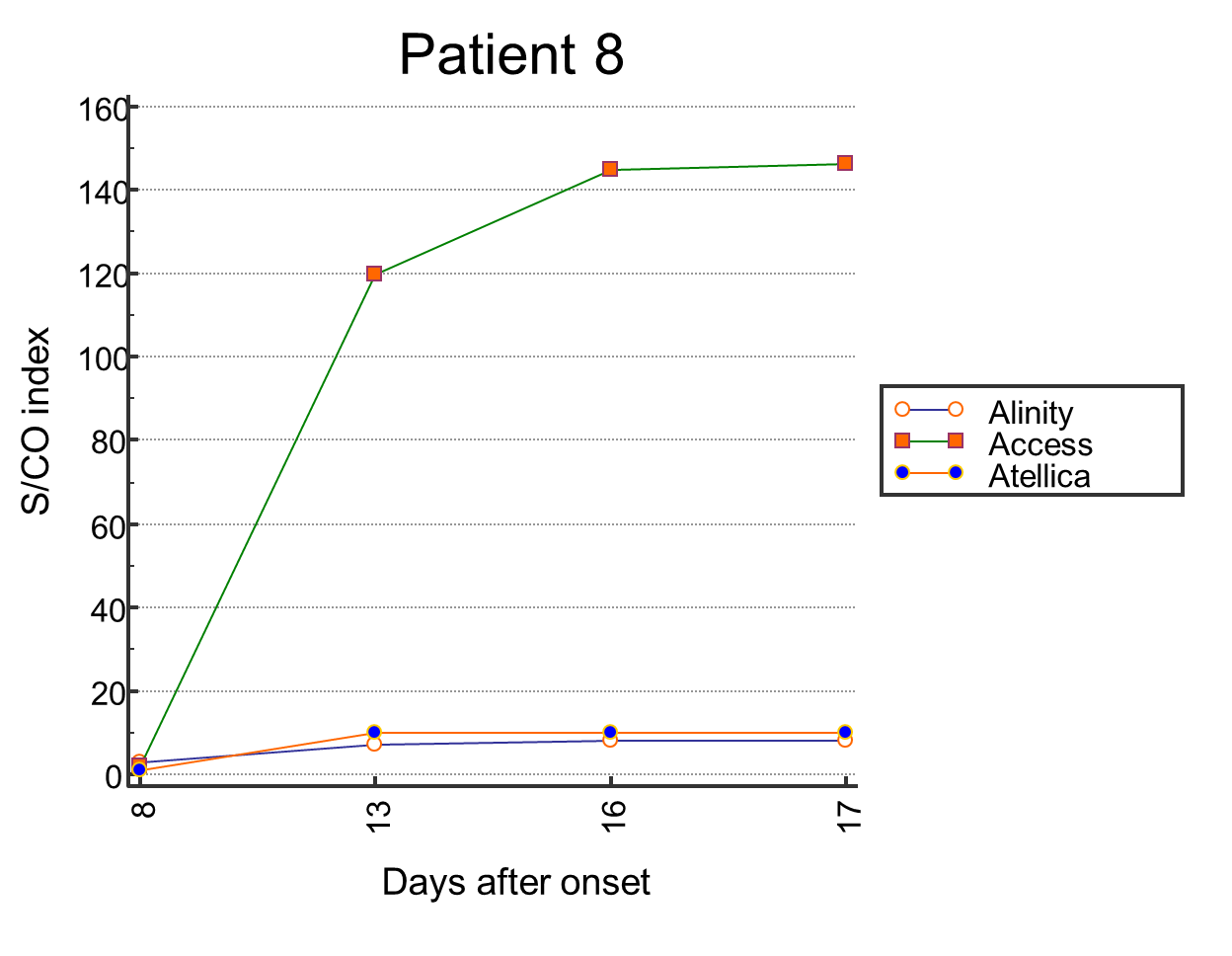

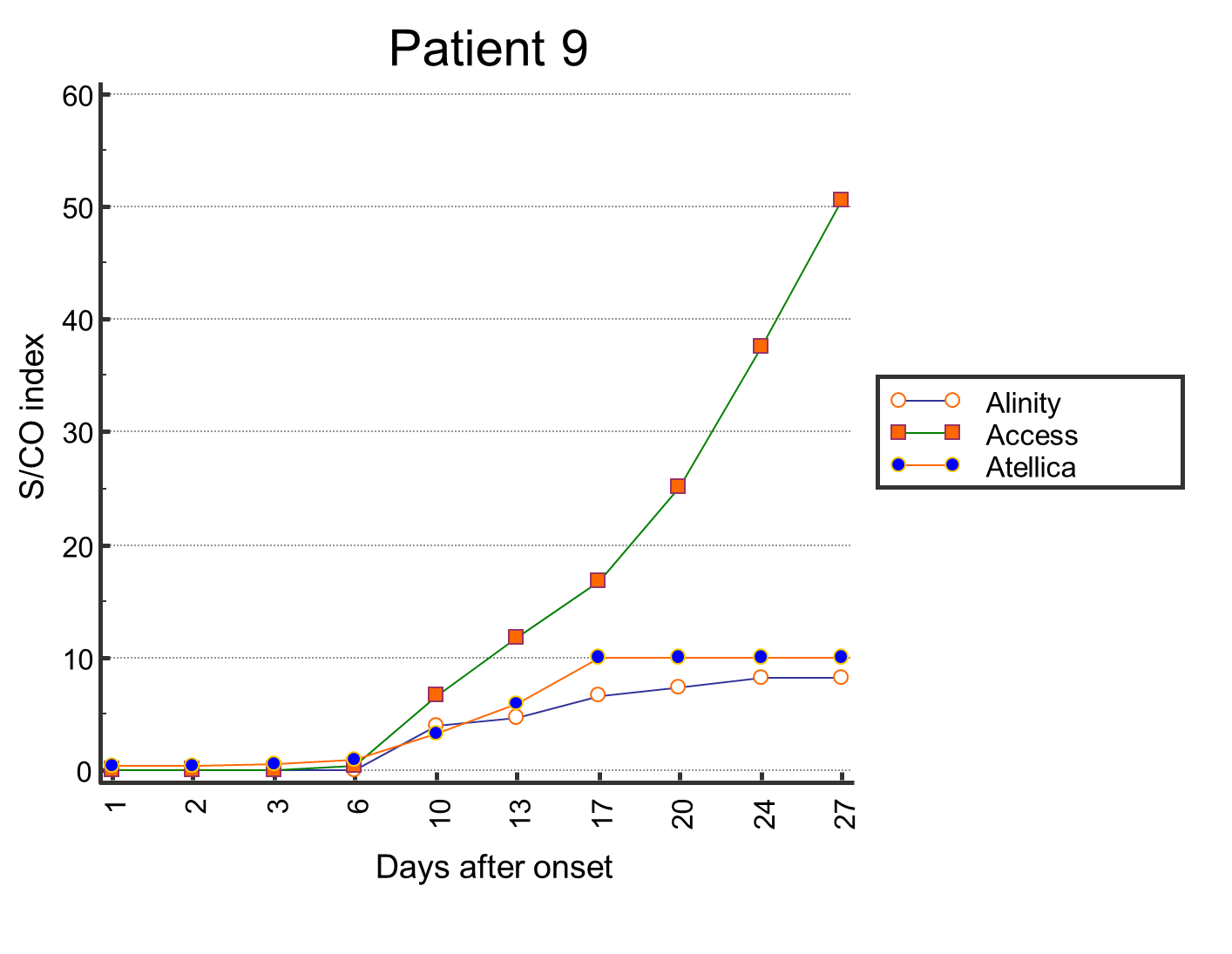

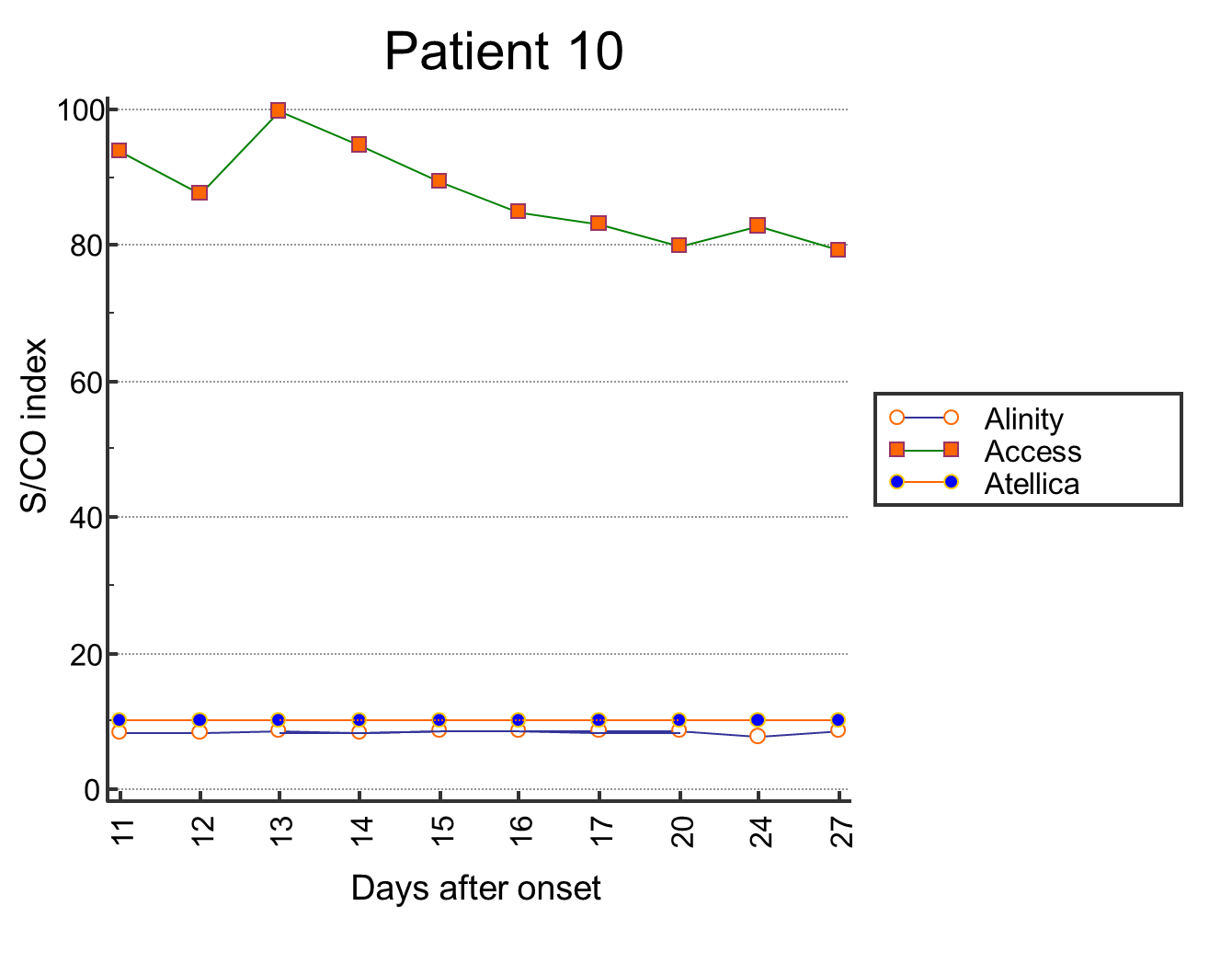

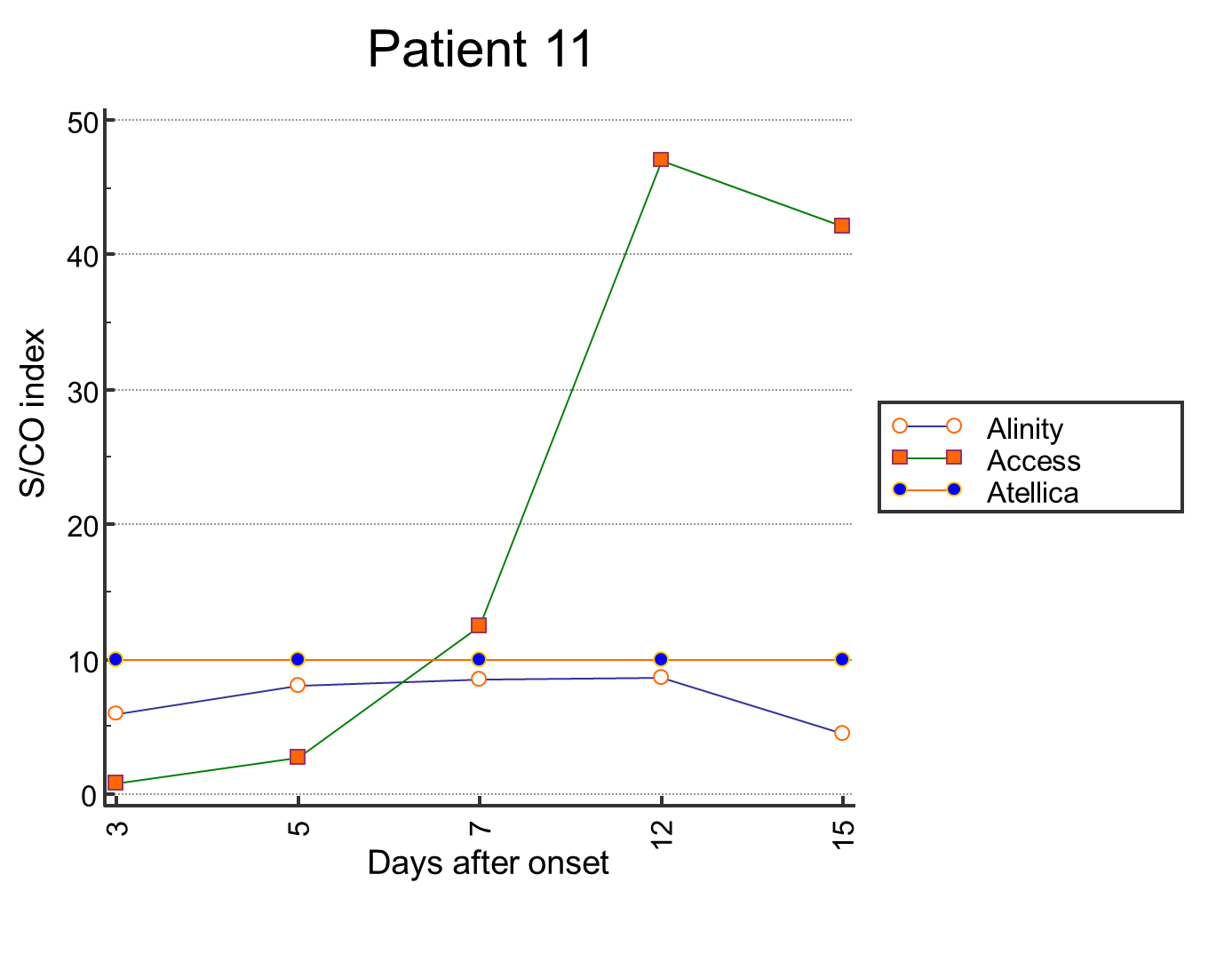

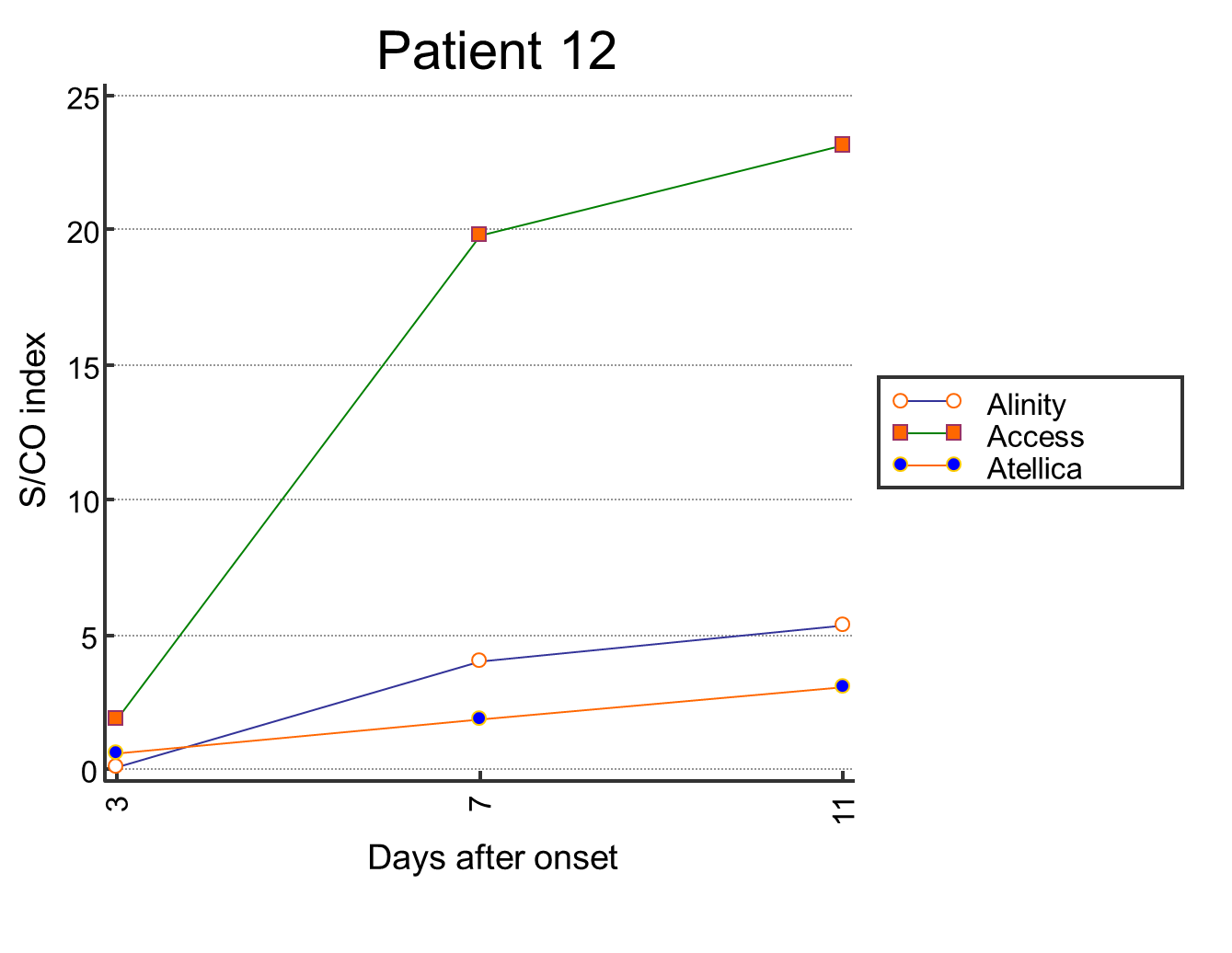

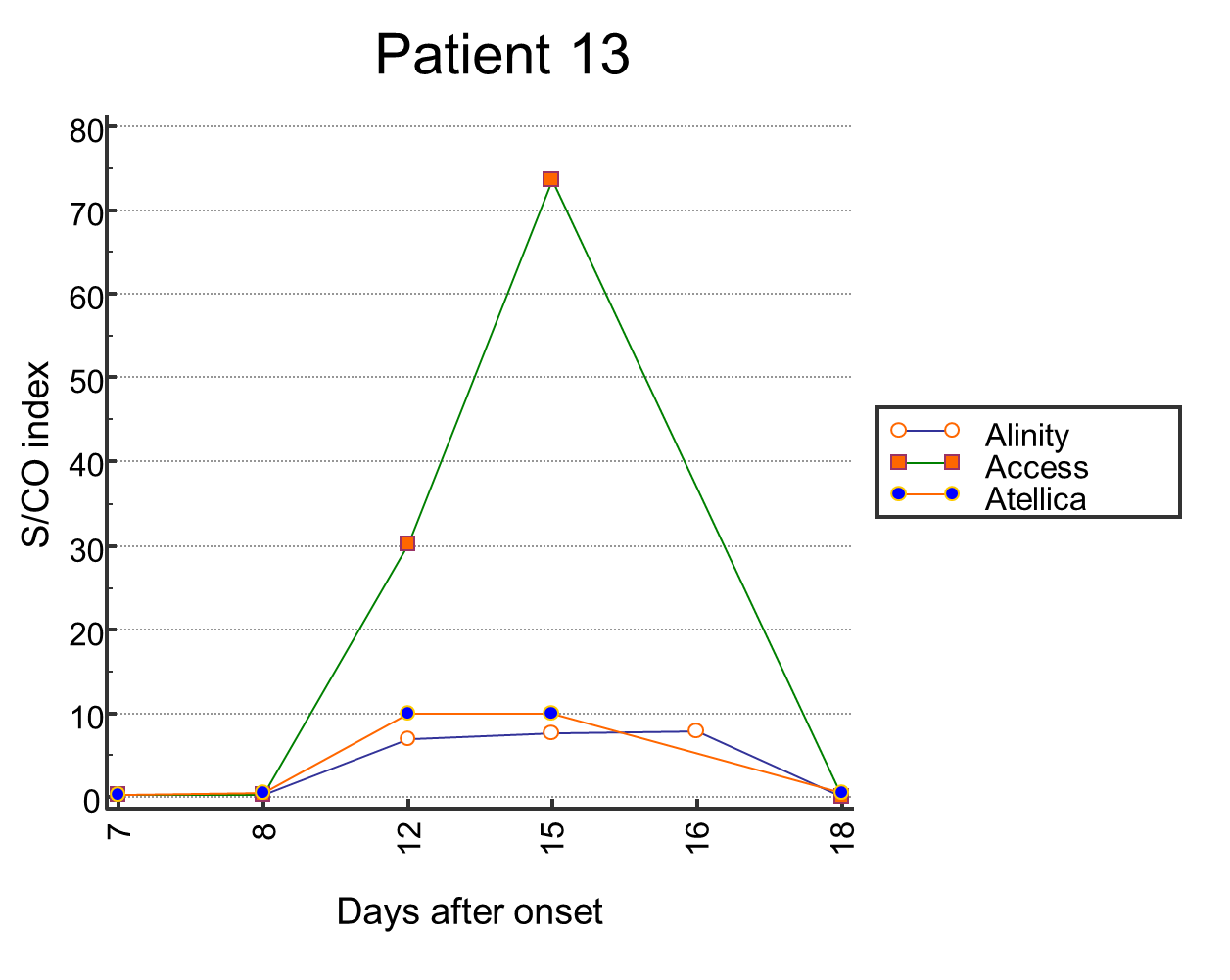

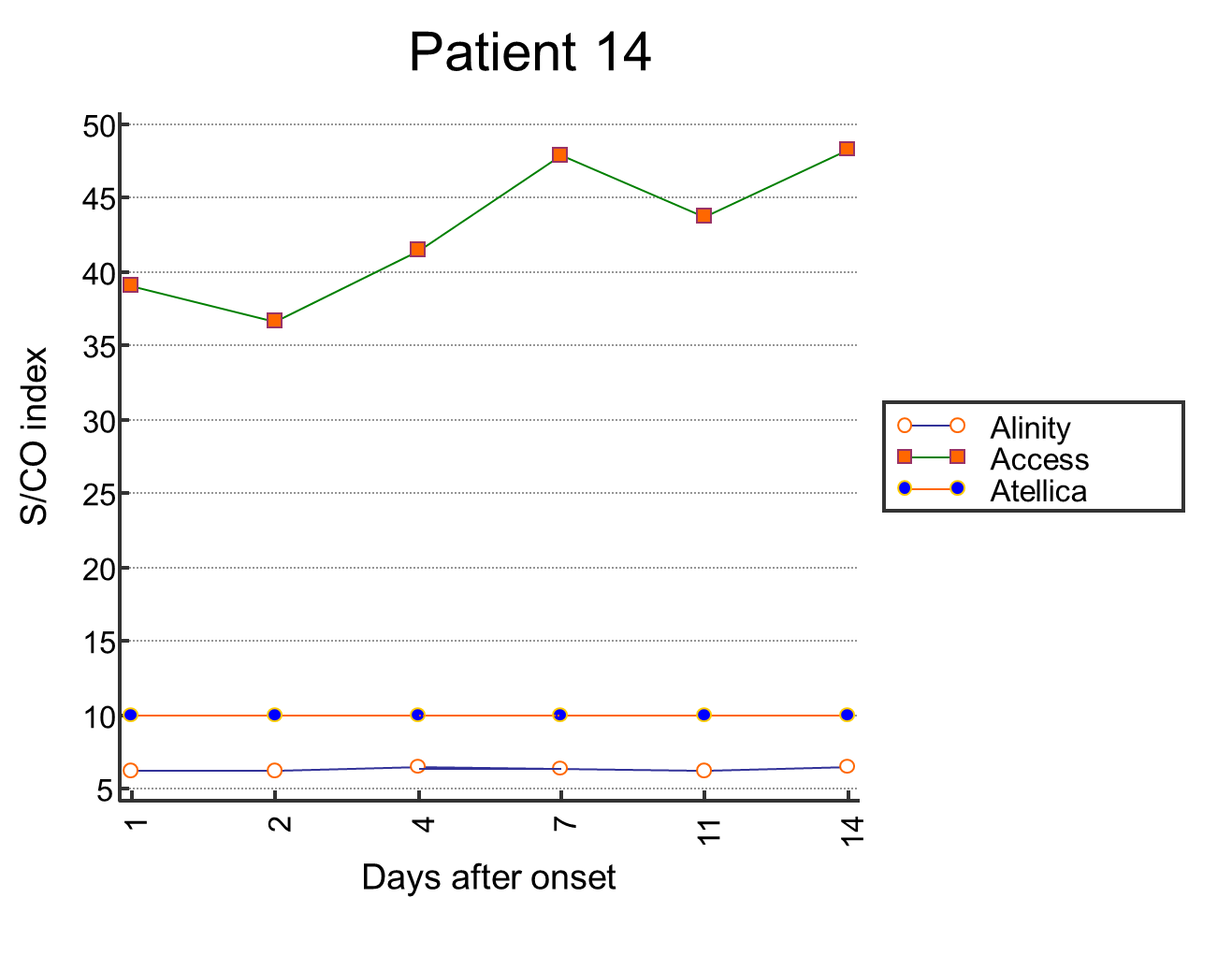

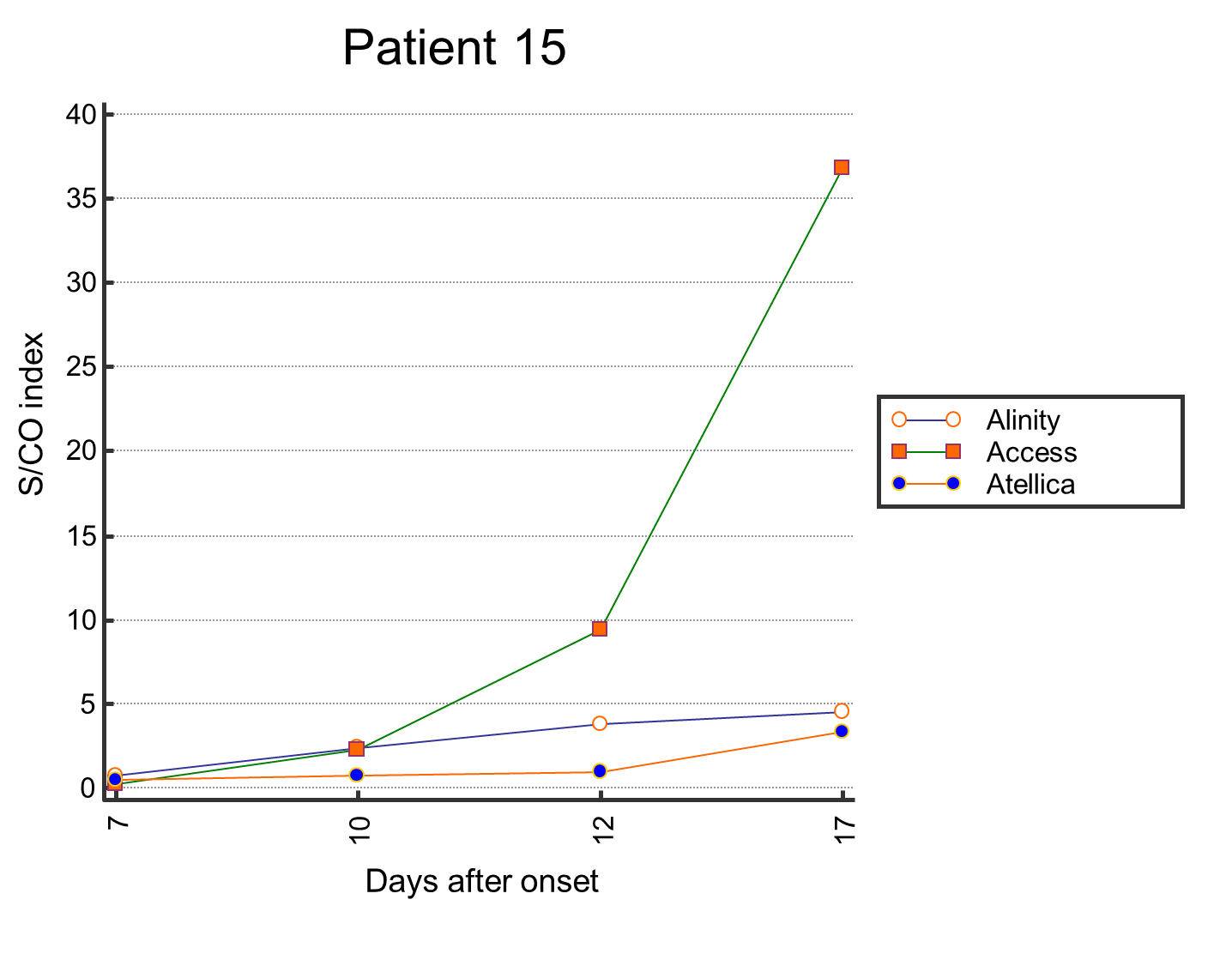

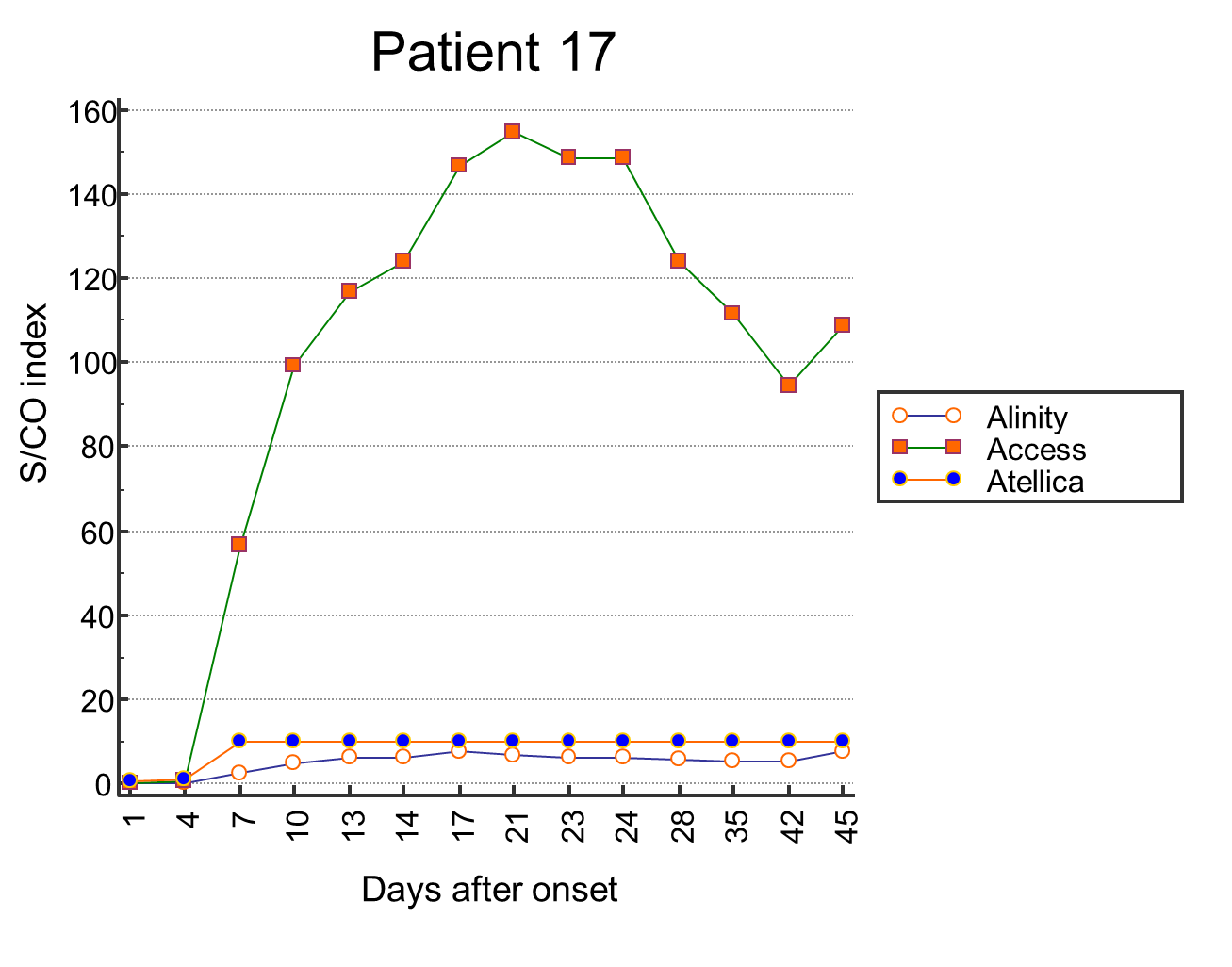

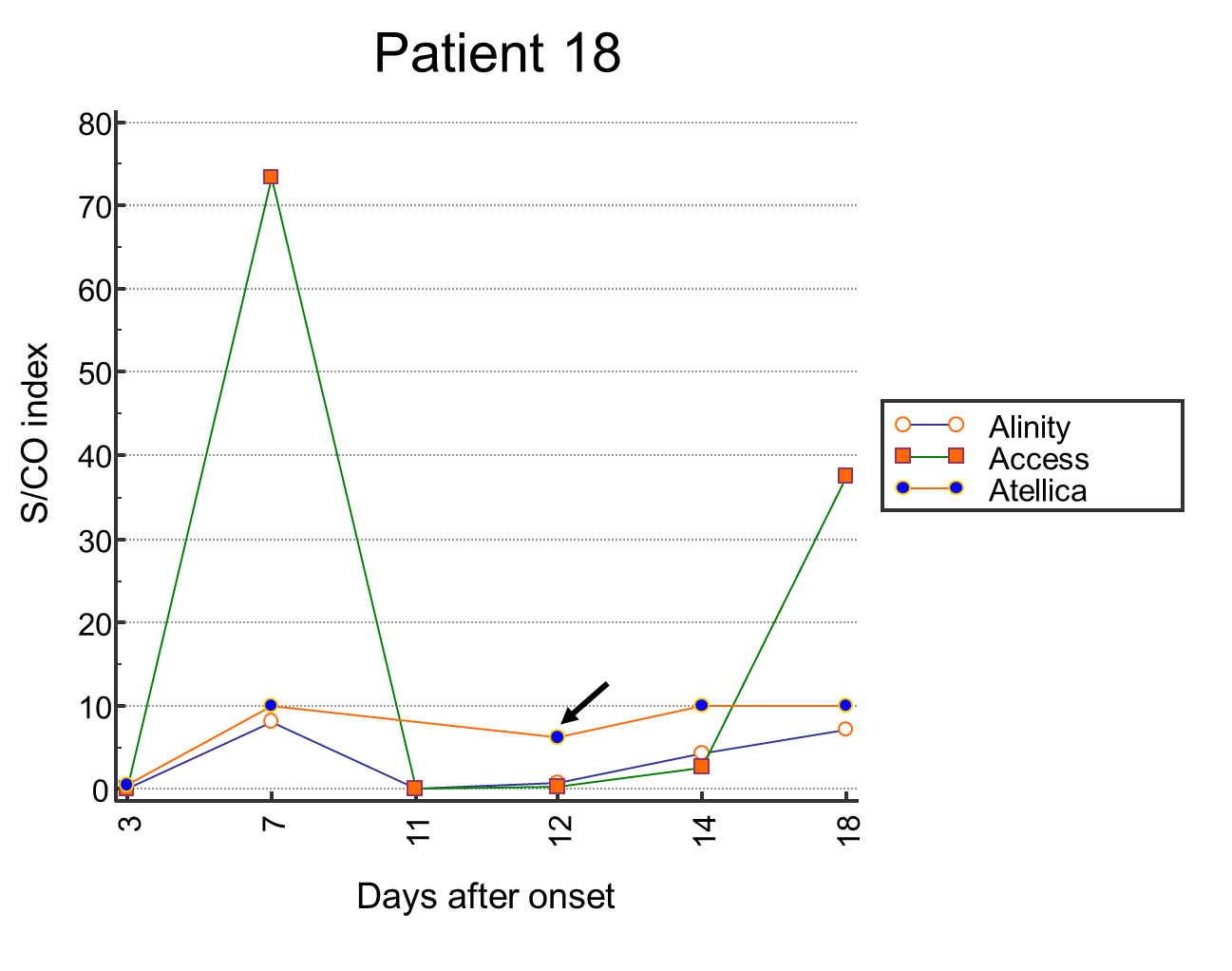

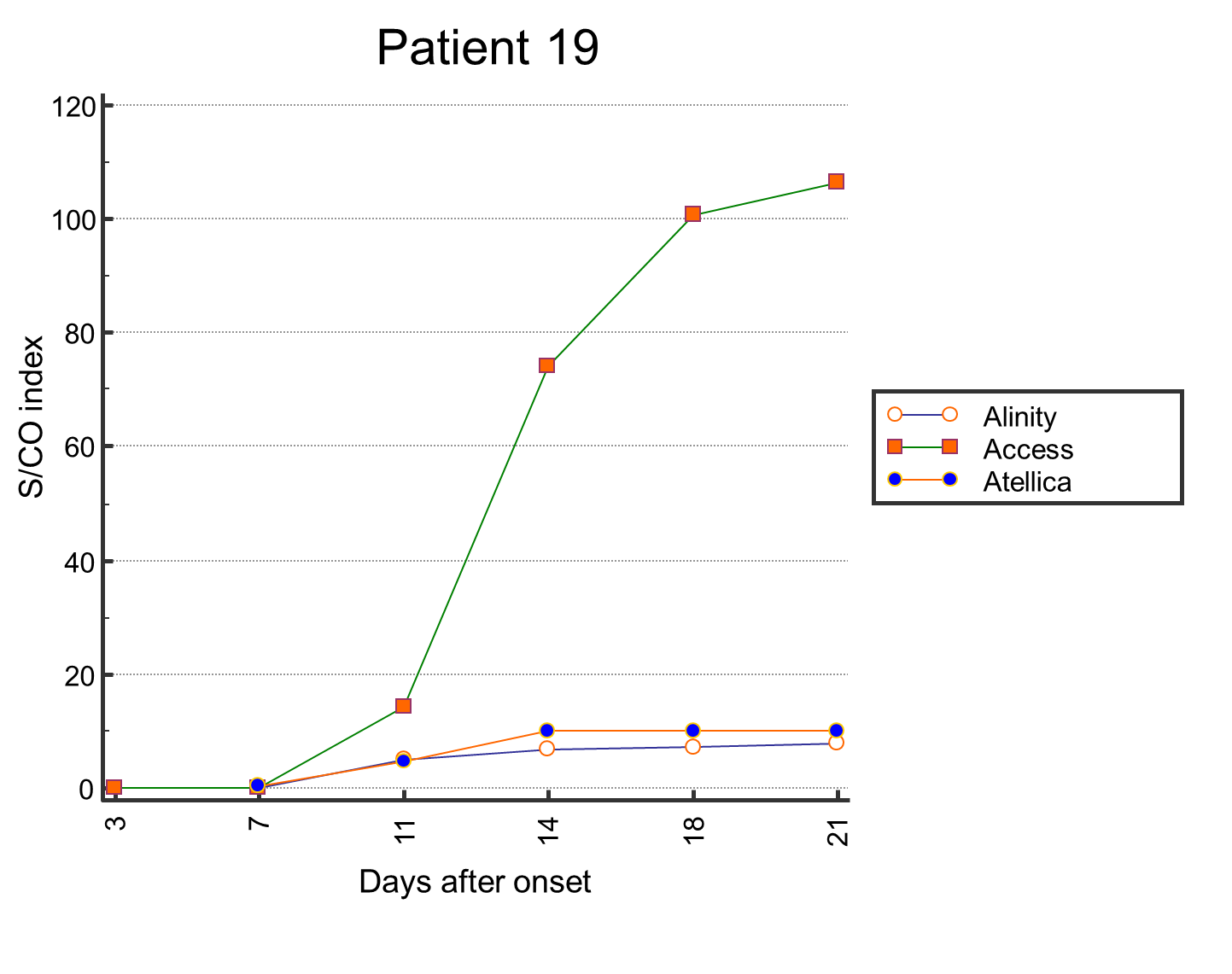

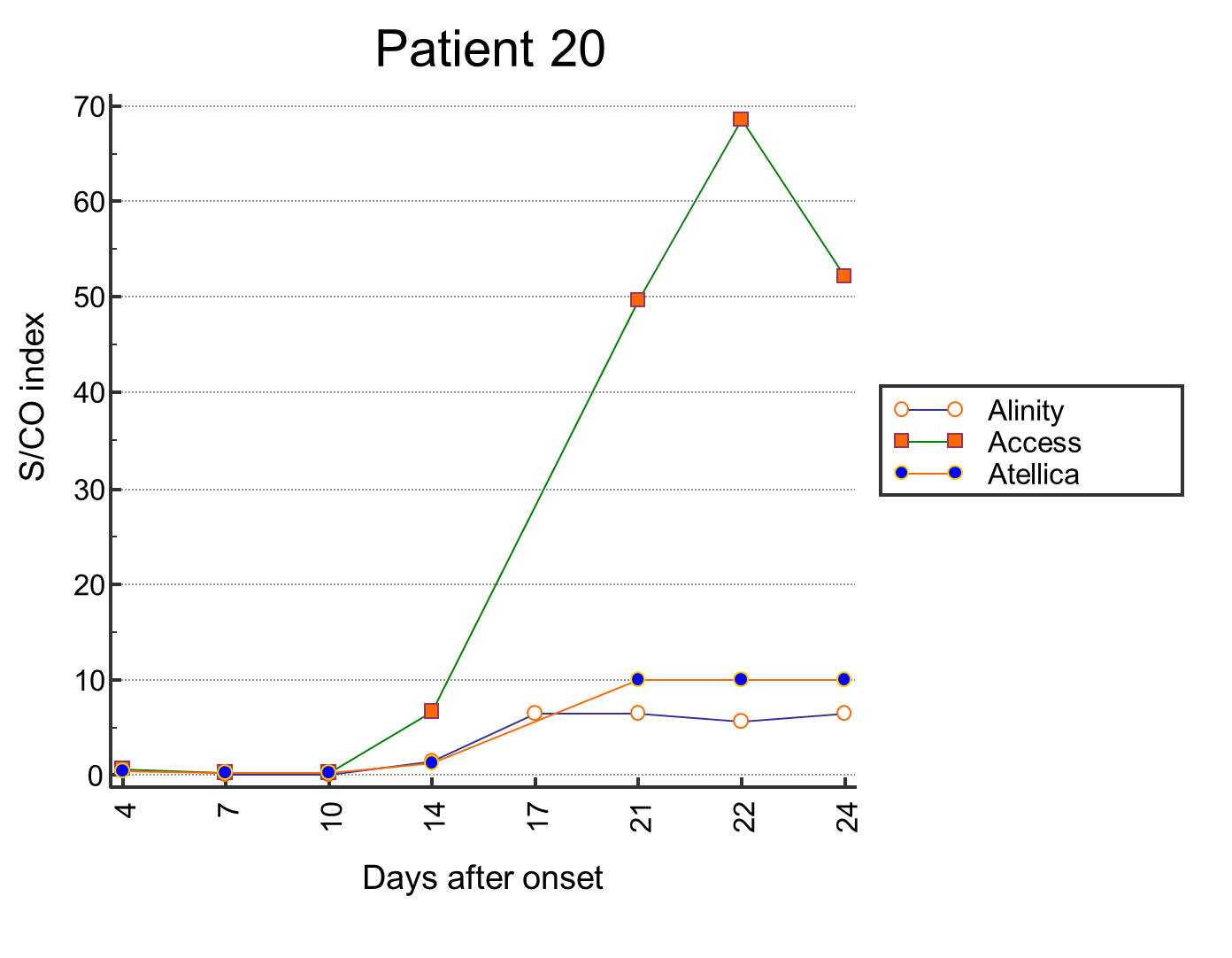

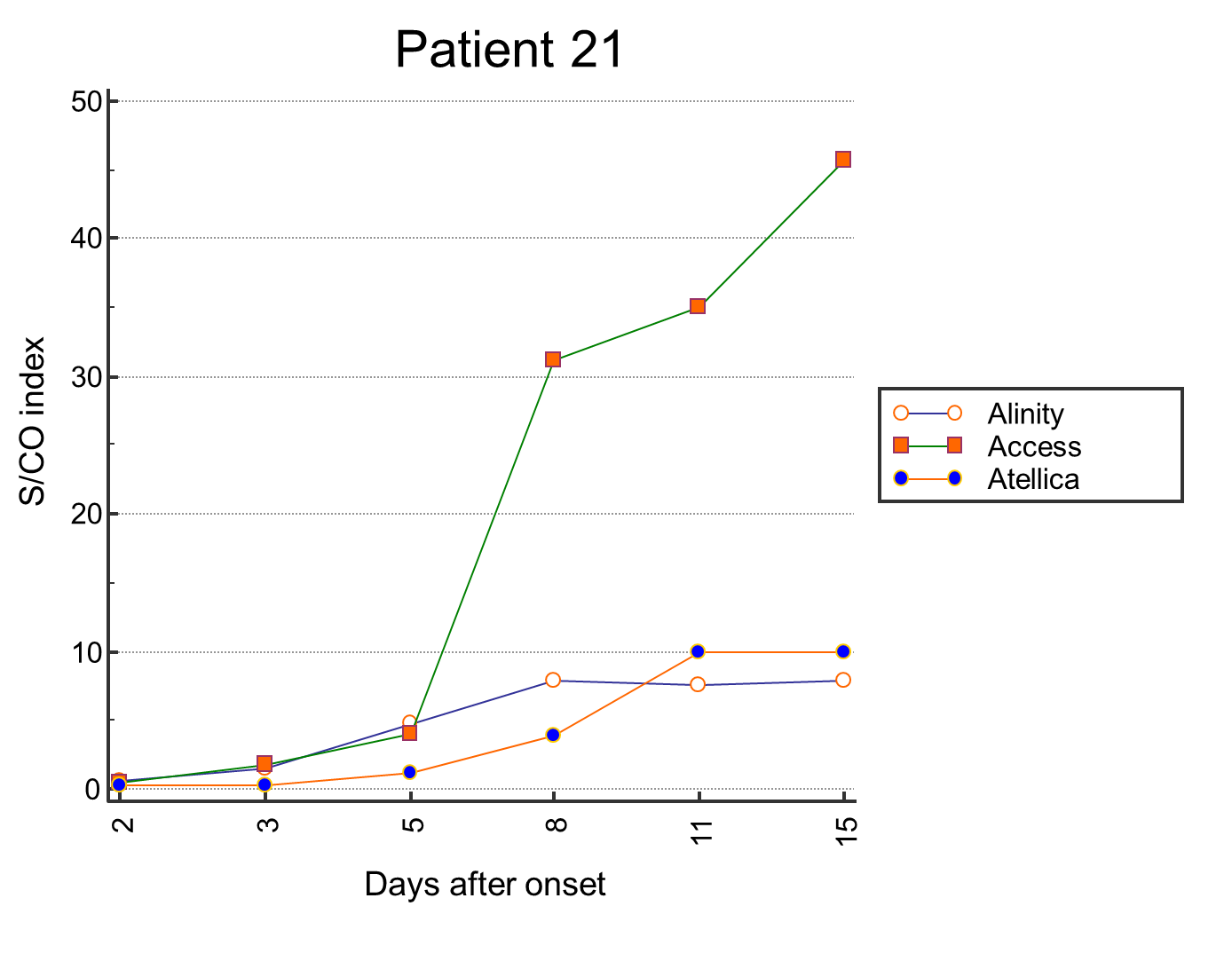

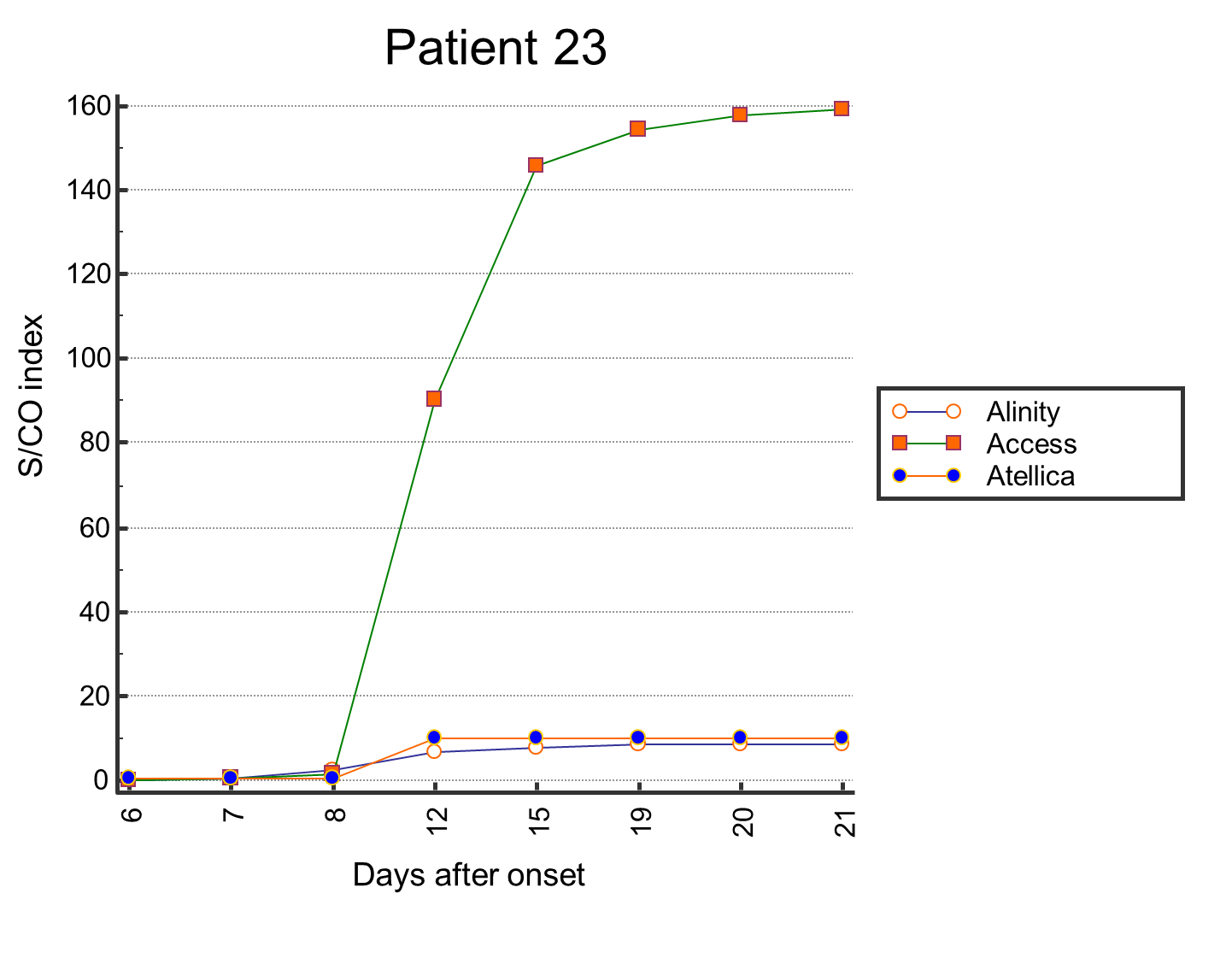

Supplement: S1 Fig — SARS-COV-2 antibody was measured by three types of automated chemiluminescence immunoassays (Alinity CoV-2 IgG, Access CoV-2 IgG, and Atellica CoV-2 Total). (DOCX) [file pone.0253889.s001.docx]
